# Plastic mulching consistently improves brinjal performance, but drip irrigation benefits are soil dependent: A two-location field study

**DOI:** 10.1371/journal.pone.0360413

**Published:** 2026-10-27

**Authors:** Md. Nayan, F. M. Jamil Uddin, Rounak Islam, Pronomi Paul Sutapa, Pallab Goswami, Swapan Kumar Paul, Tamara Jackson, Md. Harun Rashid

**Affiliations:** 1 Department of Agronomy, Bangladesh Agricultural University, Mymensingh, Bangladesh; 2 RDRS-Bangladesh, Rangpur, Bangladesh; 3 School of Agriculture, Food and Wine, Adelaide University, Adelaide, Australia; Dev Bhoomi Uttarakhand University, INDIA

## Abstract

Brinjal (*Solanum melongena* L.) requires considerable soil moisture throughout its life cycle, therefore, its cultivation in water constraint area is difficult. Though different soil moisture conservation technologies and lower water use irrigation systems have been developed for crop cultivation, their successful deployment largely depends on the locality, especially on soil characteristics. This study evaluated the effects of irrigation regimes, mulching practices, and their combination on growth, stress physiology and irrigation water use efficiency (IWUE) of brinjal across two field locations with contrasting soil conditions. Location 1 had coarser soil, lower nutrient and organic matter content profiles and lower water-holding capacity than location 2. The experiment comprised three irrigation levels [rainfed (*I*_*0*_), conventional furrow irrigation (*I*_*C*_), and drip irrigation (*I*_*D*_)] and three mulching treatments [no mulch (*M*_*0*_), straw mulch (*M*_*S*_), and plastic mulch (*M*_*P*_)] with three replications at both locations. Irrigation and mulching both significantly influenced growth, stress indicators, fruit yield, and IWUE, though the effects of the treatment on these parameters varied between locations. At Location 1, drip irrigation (*I*_*D*_) produced a fruit yield of 8.90 ± 5.68 t ha^−1^, with considerable variability among replicates, and did not differ significantly (*p* = 0.88) from conventional furrow irrigation (*I*_*C*_) or the rainfed treatment (*I*_*0*_). In contrast, at location 2, drip irrigation outperformed (21.88 ± 9.44 t ha^−1^) other treatments, being 117% and 32% higher than *I*_*0*_ and *I*_*C*_*,* respectively. Plastic mulching (*M*_*P*_) improved growth, reduced drought stress and increased yield compared to *M*_*S*_ and *M*_*0*_ at both locations. The highest yield at location 1 (23.18 ± 6.62 t ha^−1^) occurred under *I*_*C*_ *× MP*, while the highest yield at location 2 (32.93 ± 1.62 t ha^−1^) was achieved under *I*_*D*_ *× MP*. IWUE peaked at 38.84 kg ha^−1^ mm^−1^ (*I*_*C*_ *× MP*) in location 1 and 65.87 kg ha^−1^ mm^−1^ (*I*_*D*_ *× MP*) in location 2. A linear mixed model estimated that plastic mulch increased yield by ~10.63 t ha^−1^ (95% CI: 6.58–14.68 t ha^−1^, p < 0.001) over no mulch, and *I*_*C*_ *× MP* produced a synergistic yield gain of 11.03 t ha^−1^ (95% CI: 5.3–16.76 t ha^−1^, *p* = 0.003) over the control. Overall, plastic mulching consistently enhanced brinjal performance, while the benefit of drip irrigation was soil-dependent and was most evident in the higher water-retention field.

## Introduction

Water scarcity is one of most widely recognized agricultural constraints worldwide, hindering sustainable crop production [1,2]. Nearly 70% global water is withdrawn and used for crop production purposes, which will ultimately need to increase efficiency to deal with increasing population, extreme climatic conditions and urbanization [3]. Globally, agriculture is facing constant threats due to changing climate and constant variation in growing conditions [4]. Efficient water management thus becomes a top priority, especially in arid and semi-arid regions where erratic rainfall and high evaporation rate conditions prevails. In such conditions increasing water use efficiency and improving water saving technologies are crucial strategies to improve food security and productivity.

Brinjal is one of the most important household farming components in south and southeast Asia due to its role in dietary diversification, nutritional value and good income generation. Despite the high potential and income generation, brinjal productivity is often constrained by insufficient irrigation water and high evapotranspiration [5].

Regional studies showed a wide range of water requirements for brinjal in South Asia. In semi-arid field conditions of western India, full-season drip irrigation at 100% crop evapotranspiration (ETc) needed 676–691 mm, while 60–80% ETc irrigation required 405–553 mm [6]. In Bangladesh, the low-pressure and sensor-based drip irrigation systems reduced water consumption by approximately 38–63% compared to traditional farming practices (conventional furrow irrigation), while fruit yield was similar or slightly higher, and water productivity was significantly improved [7]. It was found that the irrigation requirements of brinjal are largely governed by soil water holding capacity, climate, crop duration, irrigation method and scheduling criteria [8].

Conventional flood irrigation is a widely used method for supplying water in crop production, however it causes high water loss reducing water use efficiency [3,9]. In addition to water loss, conventional irrigation, if not managed well, can cause waterlogging conditions which may put the plants under abiotic stress [10]. Water logging reduces soil quality through heavy metal deposition [11], nutrient leaching [12,13] and changing soil pH [14]. Prolonged water stagnation may aggravate soil-borne disease and insect incidence [15,16], weed infestation [17] and consequently it compromises the yield and quality of the crop produce [18]. Suitable irrigation methods, proper irrigation scheduling, and soil moisture conservation technologies, on the other hand, can mitigate these problems and create favorable conditions for crop growth under water constrained cropping systems.

Drip irrigation maintains stable soil water content, and increases water productivity and yield compared to conventional furrow and flood irrigations [19,20]. This technology, therefore, tends to improve economic benefits by lowering ongoing input costs and net carbon emission rates [21], although it does have high initial capital costs [22].

Mulching positively effects soil moisture content, reduces water loss through evaporation and improves soil organic matter [23]. Use of crop residues as mulching improves physical and chemical properties, including bulk density and aggregate stability [24]. Mulching especially plastic and straw mulching increases soil organic carbon, total nitrogen, microbial biomass carbon and nitrogen, dissolved organic carbon, water use efficiency and crop yield compare to not mulching [25]. All types of mulching effectively conserve soil moisture, soil heat and improve yield [26].

Micro-irrigation when combined with mulching optimizes fine roots of plants, improves water uptake, water use efficiency and soil water content (SWC), and at the same time reduces the SWC fluctuations [27]. This combination also improves the microclimate in the plant rootzone [28]. A combination of drip irrigation and mulching in brinjal has been found to improve water use efficiency, fruit size and yield of brinjal in previous studies [29,30]. Drip irrigation with mulching boosts water and nutrient efficiency in turmeric [31]. The combination of these two water saving technologies has also been shown to ensure early picking and higher yield of capsicum in comparison to flood irrigation and no mulch [32].

Even though drip irrigation is efficient in conserving soil moisture, its efficiency also depends on soil conditions [33, 34]. For example, Yi, Li [35] reported that frequent small volume irrigation rather than a few large volume irrigation is more efficient, especially in sandy soil for maize cultivation. Though mulching has been reported to increase soil quality [36], especially those of organic origins, understanding of their performance on different types of soil is limited. However, its performance is also likely to vary depending on the soil condition for the same reasons as in the drip irrigation.

While a large volume of research has been conducted in Bangladesh on the effects of drip irrigation, mulching and their combinations on brinjal and other crops, there is very little information on how effective these practices are in sites with different soil textures. Therefore, this study provides a preliminary comparison of their effects at two locations differing in soil physical and fertility characteristics. The field experiment was carried out to (i) determine the effect of mulching and irrigation regimes on the growth and yield of brinjal, (ii) to assess if there any synergies between mulching and irrigation treatments in terms water use efficiency, and growth and yield of brinjal, (iii) to investigate whether the effects of sole and combined effects of mulching and irrigation regimes on the performance of brinjal show similar trends across field conditions. This study also aims to estimate the effects of mulching and irrigation regimes through a linear mixed model (LMM).

## Materials and methods

### Study locations

The experiment was conducted in two locations (Fig 1) at Bangladesh Agricultural University during the period November 2024 to May 2025. The experimental sites had contrasting soil characteristics (Table 1). Location 2 had a reduced EC value (1062 µS cm^-1^) which minimizes osmotic pressure compared to location 1 (1200 µS cm^-1^). Higher values of organic carbon, nitrogen, phosphorus and potassium, the key nutrients for brinjal growth, were estimated from location 2 compared to location 1. This indicates that location 2 had better soil fertility which makes it more suited for crop production. Location 2 has a silt loam soil which means better water holding capacity compared to the sandy loam of location 1. The slightly lower particle density in location 2 is supposed to improve root penetration and aeration.

**Table 1:** Soil characteristics of the studied locations.

Soil parameter	Location 1	Location 2
pH	6.92	6.96
Electrical conductivity (µS cm^-1^)	1200	1062
Organic C (%)	1.624	2.342
Total nitrogen (%)	0.154	0.237
Phosphorus (ppm)	26.57	31.65
Potassium (cmol kg^-1^ soil)	0.147	1.126
Textural class	Sandy loam	Silt loam
Particle density (g cc^-1^)	2.79	2.63

**Fig 1:**
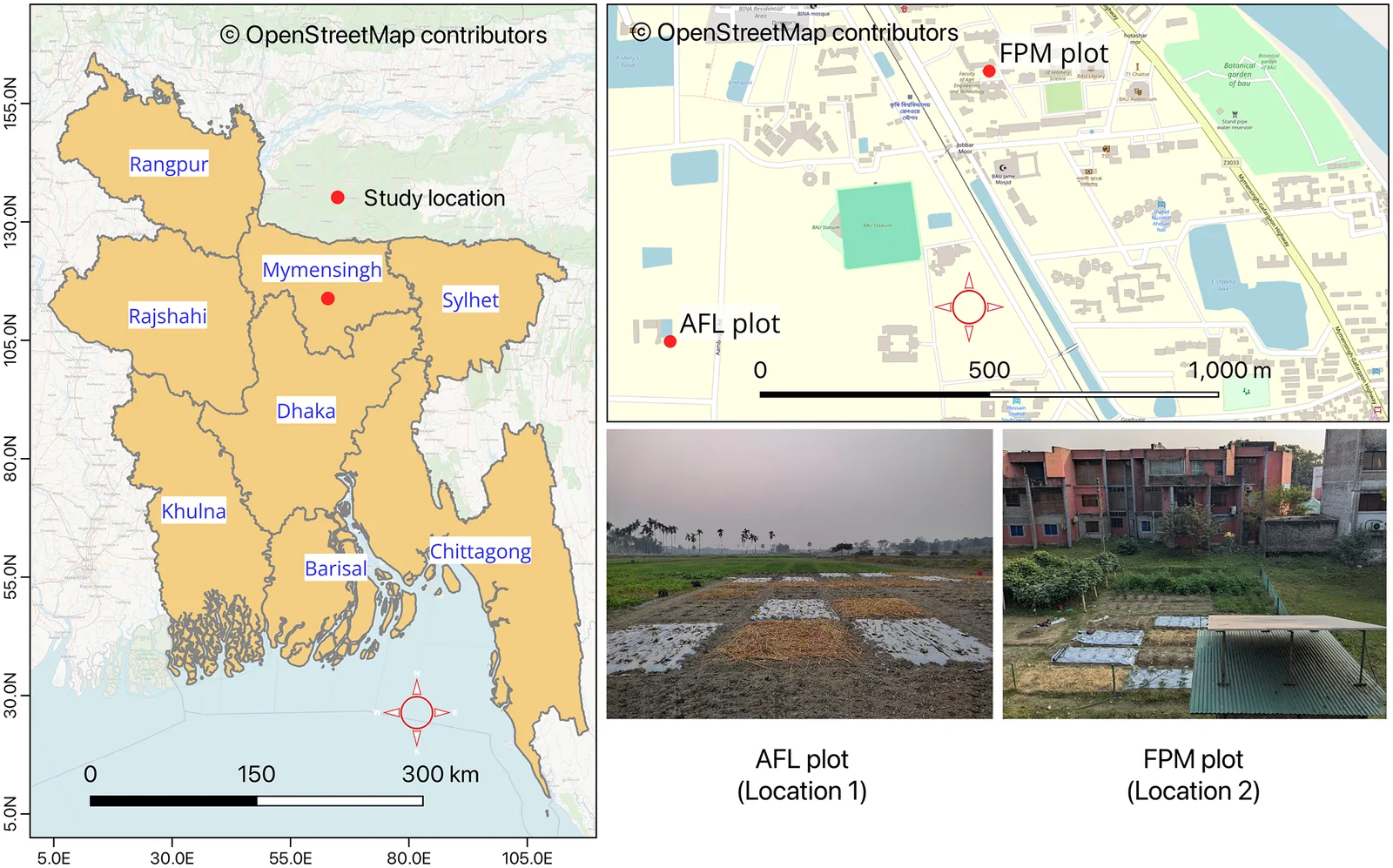
Experimental locations. Agronomy field laboratory (AFL plot: location 1) and farm power and machinery field laboratory (FPM plot: location 2) of Bangladesh Agricultural University. The map was prepared by the authors using OpenStreetMap data. Contains information from OpenStreetMap and the OpenStreetMap Foundation, made available under the Open Database License.

The experimental sites were under a subtropical monsoon climate characterized by high temperature and humidity and heavy rainfall, along with occasional wind during *kharif* (summer) season (April-September) and insufficient rainfall with moderately low temperature in *rabi* (winter) season (October-March). Rainfall in the growing period was insignificant and didn’t affect the overall production of brinjal (Fig 2).

**Fig 2:**
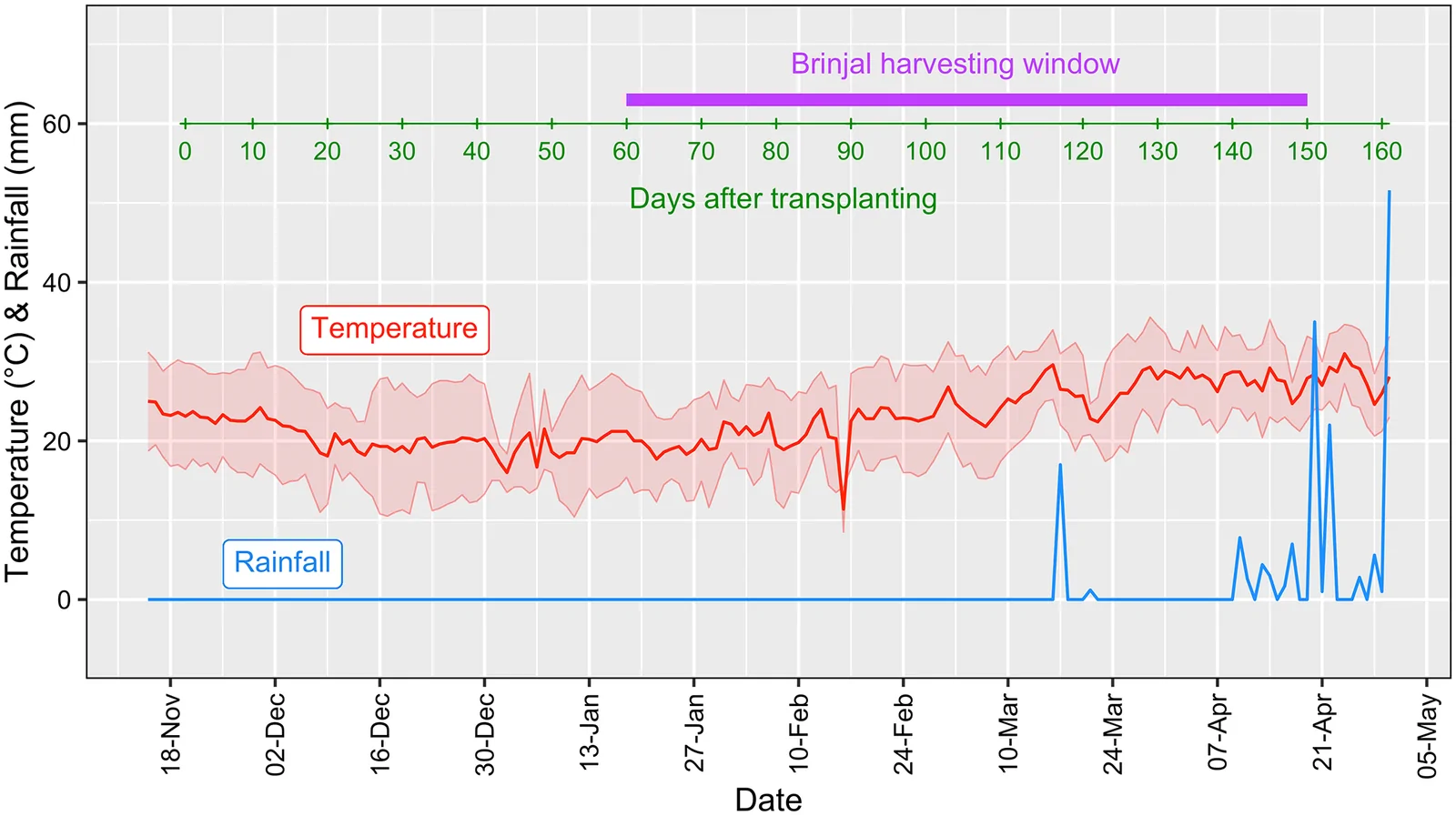
Weather conditions during the study period. The upper and lower bounds of the ribbon indicate maximum and minimum air temperature, whereas the red line is the average air temperature.

### Experimental treatments and design

Two factors were considered for this experiment, including irrigation types and application of different mulching materials. Irrigation types included three levels (i) drip irrigation (*I*_*D*_), (ii) conventional furrow irrigation (*I*_*C*_) (iii) no irrigation (rainfed condition) (*I*_*0*_), whereas mulching had three levels (i) control (no mulching) (*M*_*0*_), (ii) silver plastic mulch (*M*_*P*_) and (iii) added rice straw (*M*_*S*_). The experiment was conducted following a split-plot design, where irrigation was allocated in the main plots and mulching to the sub-plots. The total number of plots number was 27, each enclosing 2.5 m × 2 m.

### Setting up the experiment and agronomic management

The land was well ploughed, and cow dung was added at the rate of 5−6 t ha^-1^. Black and transparent mulching were bought from local markets and cut to the right area. Straw was collected from the agronomy field and before use it was sun dried for two days. The experimental plots were fertilized with urea, triple super phosphate (TSP), muriate of potash (MoP) and gypsum at a rate of 480 kg, 280 kg, 200 kg and 200 kg ha^-1^, respectively [37]. The total amount of TSP, MoP and gypsum were broadcast and incorporated into the soil at final land preparation. The first 1/3rd of urea was applied at final land preparation on 20^th^ November 2024. The rest of the urea were applied making a band around the plants in 2 installments: 1/3rd at 25 days after sowing (DAS) and the other 1/3rd at 50 DAS.

Brinjal seedlings (‘Purple King’, Lal Teer Seed Limited, Gazipur, Bangladesh) were planted on 20^th^ November 2024. Each plot had 3 rows and row to row distance was around 90 cm and plant to plant distance was 50 cm. A pit was made with shovel, and seedlings were planted in the pit. Care was taken to protect the seedlings from birds and rodents up to 20 days after planting and irrigated regularly to help the seedlings to overcome transplanting shock. In some pits, the seedlings didn’t survive. Seedlings of the same age from the same area was replaced with vigorous plants. Three weeding, one at 20, second at 60 and third at 85 days after planting were done.

Irrigation was given regularly automatically with drip irrigation system, for conventional irrigation water was applied through alternate furrow 3 times in a 20-day interval. This interval for conventional irrigation represents the less-frequent end of the traditional farmer-managed surface-irrigation practice used for brinjal production in Bangladesh. This treatment was included as a practical conventional-irrigation benchmark for comparison with drip irrigation rather than as an optimized irrigation schedule.

A locally developed gravity-fed drip irrigation system was used for drip irrigation. We did not use a commercial pump or pressure-compensated emitter. The discharge rate of the emitters was checked on 20 randomly chosen emitters prior to installation in the field. Mean emitter discharge was 3.3 ± 0.3 L h^-1^ emitter^-1^, indicating an approximate but calibrated water delivery rate.

An emitter was placed near each brinjal plant. The planting density was 22,222 plants ha^-1^. The drip system was operated for 1 h on alternate days during the crop growing period. Thus, each plant received ~3.3 L of irrigation water per irrigation event. The amount of irrigation water applied was about 73,333 L ha^-1^ per irrigation event corresponding to 7.33 mm per event, calculated based on the planting density. Irrigation was applied every second day, meaning that the average daily input of irrigation was around 36667 L ha^-1^ day^-1^ or 3.67 mm day^-1^. The total amount of seasonal irrigation water applied, through the drip system, was calculated as the product of the amount of water applied per irrigation event and the total number of irrigation events.

Bamboo stakes (1.5 m height) placed 10 cm from each plant to provide physical support to the plants. Insect- pests were regularly controlled using biological and chemical methods.

### Harvesting and data collection

Brinjal were harvested in many installments and data were assembled after the final harvesting. All the necessary data on yield contributing characters were collected from ten randomly selected and marked plants from each plot. Data on growth parameters were collected from these plants and pooled for individual plots. However, data from the whole plot was aggregated for yield estimation.

### Determination of irrigation water use efficiency

Irrigation water use efficiency of brinjal in different irrigation and mulching managements were estimated using the following equation:


$$\begin{array}{c} IWUE=\frac{Y}{TW} \end{array}$$

where, I*WUE* = Irrigation water use efficiency (kg ha^-1^ mm^-1^), *Y* = crop yield (kg ha^-1^), and *TW* = Total water applied (mm).

### Biochemical stress marker assay

Growth, yield, and irrigation water-use efficiency were evaluated at both field locations, whereas biochemical stress markers were assessed only at Location 2; therefore, physiological responses were interpreted solely for that location.

#### Hydrogen peroxide (H_2_O_2_).

Hydrogen peroxide content was determined following the method of Murshed, Lopez-Lauri [38]. In short, in an ice bath system frozen eggplant leaves (0.25 g) were homogenized using 1 ml of 0.1% (*w/v*) TCA and centrifuged at 12000 × g over 15 min at 4 °C. 100 µL from each of the tube were placed in 96-well plates, where 50 µL of 10 mm potassium phosphate buffer (pH 7.0) and 100 µL of 1 ml KI. After being vortexed the plate was incubated at room temperature for 30 minutes and absorbance were recorded at 390 nm in microplate reader. Finally, H_2_O_2_ content was measured using standard curve.

#### Malondialdehyde (MDA) content.

Lipid peroxidation was measured by the determination of malondialdehyde (MDA) following the method of Hannachi, Werbrouck [39] and Hodges, DeLong [40], where 1 g of leaf and 80% ethanol were used. MDA was determined using reaction with thiobarbituric acid (TBA). Then, the absorbances were read at 532 nm in spectrophotometer and Malondialdehyde (MDA) equivalents were estimated according to [39].

#### Catalase (CAT) content.

Catalase (CAT) activity was analyzed (EC 1.11.1.6) following [41] and was estimated by measuring the reduction of H_2_O_2_ (ε = 2.3 mM^−1^ cm^−1^) at 240 nm. CAT activity was determined with a reaction mixture of 1900 µL of potassium phosphate buffer (50 mM, pH 7.0, not containing EDTA), 100 µL sample, and 1000 µL H_2_O_2_ (30 mM) and it was expressed as U mg^−1^ of protein.

#### Ascorbate peroxidase (APX).

Ascorbate peroxidase (APX) activity (EC 1.11.1.11) was analyzed by using the methodology published by [41]. The mixture used in the reaction had contained 50 mM of potassium phosphate buffer (pH 7.0), 4.4 µL ascorbate (1 mM), and 10 µL EDTA-2Na (0.5 M). The reaction was initiated by using H_2_O_2_, ascorbate oxidation was recorded at 290 nm for 3 minutes. Activity was determined using the extinction coefficient, e = 2.8 mM^−1^ cm^−1^. Each sample was assayed in three replications. Results were expressed in U mg^−1^ of protein.

#### Proline content.

Proline was determined following the protocol adopted by Bates, Waldren [42]. Briefly, the extraction of 500 mg of plant tissue was performed using 3% (*w*/*v*) sulfosalicylic acid. The concentration of proline in the extract was determined spectrophotometrically at 520 nm using toluene as blank. Proline content was estimated as µg proline g^−1^ FW.

### Statistical analysis, modeling and visualization

All statistical analyses were performed using ‘R’ (version 4.4.1) [43] and data visualizations were prepared with the ‘ggplot2’ package of ‘R’. Factorial analysis of variance (ANOVA) was performed to judge if levels of irrigation, mulching and their interaction had any significant effect on growth, yield related parameters, yield and stress physiology of brinjal. Mean separation was carried out by Tuke’s *post hoc* test with the help of ‘agricolae’ package of ‘R’. ANOVA was performed separately on the data from location 1 (agronomy field laboratory) and location 2 (engineering field). Before performing ANOVA on each data set, the assumption of normality was confirmed by the Shapiro-Wilk test. The outcome of the analysis and their visualization are presented separately. To model the effect of irrigation and mulching on brinjal fruit yield, linear mixed-effect models (LMMs) [44] were deployed. The ‘lmer’ function of the packages ‘lme4’ using REML and ‘nloptwrap’ optimizer [44] of ‘R’ was used in the models. The following three models were considered:

Model 1: to make a general inference across locations


$$\begin{array}{c} y\sim irrigation*mulch+(1|location/replication) \end{array}$$

Model 2: to make location-specific inference


$$\begin{array}{c} y\sim irrigation*mulch*location+(1|replication) \end{array}$$

Model 3: to test if the treatment effects differ across locations


$$\begin{array}{c} y\sim irrigation*mulch+(1+irrigation*mulch|location) \end{array}$$

Among the three candidate models, model 2 was selected based on its Akaike information criterion (AIC) and log Likelihood (logLik) and likelihood ratio test (Table 2). The lowest AIC value, the highest logLik value and a significant likelihood ratio test determined the best model.

**Table 2:** Selection of the best model from three candidate models based on AIC, logLik and likelihood ratio test.

	npar	AIC	BIC	logLik	Deviance	Chisq	df	*p*-value
Model1	12	338.7	362.57	−157.35	314.7			
Model2^†^	20	291.88	331.66	−125.94	251.88	62.817	8	<0.001***
Model3	55	373.04	482.43	−131.52	263.04	0	35	

† Best model

Standardized parameters were obtained by fitting the model on a standardized version of the dataset. 95% Confidence Intervals (CIs) and p-values were computed using a Wald t-distribution approximation. Three models were tested and verified posteriori for heteroscedasticity and overdispersion of residuals from the best model (‘testDispersion’ function of the R package ‘DHARMa’) [45].

## Results

### Plant growth

#### Leaf greenness.

At location 1, leaf greenness (SPAD value) was significantly influenced by irrigation. Highest SPAD value was observed under *I*_*0*_ (85.8 ± 17.66), which was statistically similar with *I*_*D*_ (84.58 ± 13.3). The lowest SPAD value was observed under *I*_*C*_ (64.3 ± 16.61) (Table 3). On the contrary, irrigation did not show any significant effect at location 2 (Table 4). Mulching significantly improved SPAD value. Highest SPAD value (89.46 ± 14.54) was observed in *M*_*P*_ and lowest (69.04 ± 9.48) was found in *M*_*0*_, which was statistically similar with *M*_*S*_ (76.18 ± 23.45) (Table 3). At location 2, a similar trend was observed. The highest value (93.3 ± 11.65) was found in *M*_*P*_ and lowest (70.63 ± 12.95) was found in *M*_*0*_ treatment. Both were statistically similar with *M*_*S*_ (77.91 ± 18.24) (Table 4). At location 1, the interaction between irrigation and mulching significantly influenced the SPAD value. The highest SPAD value (100.5 ± 13.86) was observed under *I*_*0*_ *× M*_*P*_, which was statistically similar with all the treatments except *I*_*0*_ *× M*_*0*_, *I*_*C*_ *× M*_*0*_ and *I*_*C*_ *× M*_*S*_. The lowest SPAD value (46.17 ± 4.19) occurred in the *I*_*C*_ *× M*_*S*_, which was statistically similar with *I*_*0*_ *× M*_*0*_ and *I*_*C*_ *× M*_*0*_ (Table 3). At location 2, the interaction effect on SPAD value was not statistically significant (Table 4).

**Table 3:** Effect of irrigation, mulch and their interaction on growth and yield related traits of brinjal at location 1.

Treatment	SPAD value	Plant height (cm)	Leaf area index (LAI)
Irrigation (I)			
*I*_*0*_	85.8 ± 17.66 a	67.14 ± 6.85	118.01 ± 14.61 b
*I*_*C*_	64.3 ± 16.61 b	62.21 ± 6.07	125.11 ± 23.65 ab
*I*_*D*_	84.58 ± 13.3 a	67.63 ± 5.53	129.23 ± 14.3 a
Sig. level	**	ns	**
Mulching (M)			
*M*_*0*_	69.04 ± 9.48 b	60.47 ± 4.2 b	105.56 ± 5.8 c
*M*_*P*_	89.46 ± 14.54 a	69.92 ± 6.26 a	143.13 ± 10.67 a
*M*_*S*_	76.18 ± 23.45 b	66.59 ± 5.11 a	123.66 ± 10.38 b
Sig. level	**	**	**
Interaction (I × M)			
*I*_*0*_ *× M*_*0*_	66.3 ± 5.43 bc	60.09 ± 3.1	104.15 ± 3.48 de
*I*_*0*_ *× M*_*P*_	100.5 ± 13.86 a	73.66 ± 5.02	134.81 ± 8.69 bc
*I*_*0*_ *× M*_*S*_	90.6 ± 9.81 ab	67.67 ± 3.79	115.06 ± 6.51 de
*I*_*C*_ *× M*_*0*_	68.17 ± 15.96 bc	57.66 ± 5.22	101.65 ± 6.03 e
*I*_*C*_ *× M*_*P*_	78.57 ± 3.26 ab	66.43 ± 4.87	153.61 ± 9.66 a
*I*_*C*_ *× M*_*S*_	46.17 ± 4.19 c	62.55 ± 6.21	120.07 ± 5.09 cd
*I*_*D*_ *× M*_*0*_	72.67 ± 6.55 abc	63.67 ± 2.52	110.87 ± 4.24 de
*I*_*D*_ *× M*_*P*_	89.3 ± 16.8 ab	69.66 ± 8.27	140.97 ± 3.38 ab
*I*_*D*_ *× M*_*S*_	91.77 ± 7.67 ab	69.56 ± 3.5	135.86 ± 3.09 bc
Sig. level	**	ns	**
CV (%)	13.37	7.63	4.84

Values are mean ± standard deviation of means; I0: no irrigation (rainfed), *I*_*C*_: conventional furrow irrigation, *I*_*D*_: drip irrigation; M0: no mulching, *M*_*P*_: plastic mulching, *M*_*S*_: straw mulching; and designate significant at 99% and 95% level of significance; NS: not significant: CV: co-efficient of variation; Means within a column followed by the same letter or without a letter are not significantly different at the 5% probability level according to Tukey’s HSD test. ** indicates significance at *p* < 0.01; ns indicates a nonsignificant effect (*p* ≥ 0.05).

**Table 4:** Effect of irrigation, mulch and their interaction on growth and yield related traits of brinjal at location 2.

Treatment	SPAD value	Plant height (cm)	Leaf area index (LAI)
Irrigation (I)			
*I*_*0*_	81.27 ± 14.59	67.14 ± 6.85 b	86.00 ± 11.98 c
*I*_*C*_	78.66 ± 17.53	91.22 ± 16.15 a	104.21 ± 19.79 b
*I*_*D*_	77.39 ± 16.49	94.89 ± 27.05 a	125.85 ± 15.35 a
Sig. level	ns	*	**
Mulching (M)			
*M*_*0*_	70.63 ± 12.95 b	77.02 ± 20.48	94.67 ± 18.37 b
*M*_*P*_	93.3 ± 11.65 a	92.74 ± 22.69	119.01 ± 23.75 a
*M*_*S*_	77.91 ± 18.24 ab	83.48 ± 21.82	102.39 ± 20.42 b
Sig. level	**	ns	**
Interaction (I × M)			
*I*_*0*_ *× M*_*0*_	66.3 ± 5.43	60.09 ± 3.1	76.79 ± 4.75
*I*_*0*_ *× M*_*P*_	100.5 ± 13.86	73.66 ± 5.02	100 ± 9.54
*I*_*0*_ *× M*_*S*_	90.6 ± 9.81	67.67 ± 3.79	81.21 ± 2.22
*I*_*C*_ *× M*_*0*_	63.47 ± 3.09	84.87 ± 18.37	93.13 ± 13.09
*I*_*C*_ *× M*_*P*_	97.5 ± 6.55	99.45 ± 17.82	117.85 ± 27.45
*I*_*C*_ *× M*_*S*_	75 ± 16.67	89.33 ± 14.84	101.67 ± 13.01
*I*_*D*_ *× M*_*0*_	82.13 ± 18.1	86.11 ± 26.16	114.09 ± 10.36
*I*_*D*_ *× M*_*P*_	81.9 ± 3.04	105.11 ± 29.54	139.18 ± 16.03
*I*_*D*_ *× M*_*S*_	68.13 ± 23.63	93.43 ± 33.09	124.29 ± 10
Sig. level	ns	ns	ns
CV (%)	16.25	23.61	12.96

Values are mean ± standard deviation of means; I0: no irrigation (rainfed), *I*_*C*_: conventional furrow irrigation, *I*_*D*_: drip irrigation; M0: no mulching, *M*_*P*_: plastic mulching, *M*_*S*_: straw mulching; and designate significant at 99% and 95% level of significance; NS: not significant: CV: co-efficient of variation; Means within a column followed by the same letter or without a letter are not significantly different at the 5% probability level according to Tukey’s HSD test. * and ** indicate significance at p < 0.05 and p < 0.01, respectively; ns indicates a nonsignificant effect (p ≥ 0.05).

#### Plant height.

At location 1, irrigation did not affect plant height significantly (Table 3). Whereas at location 2, irrigation significantly boosted plant height, *I*_*D*_ produced the tallest plant (94.89 ± 27.05 cm), which was statistically similar with *I*_*C*_ (91.22 ± 16.15 cm). The shortest plant was observed under *I*_*0*_ (67.14 ± 6.85 cm) (Table 4). Plant height was significantly improved by mulching, at location 1. Tallest plant (69.92 ± 6.26 cm) was observed in *M*_*P*_, which was statistically similar with *M*_*S*_ (66.59 ± 5.11 cm). Whereas shortest plant was observed at *M*_*0*_ (60.47 ± 4.2 cm) (Table 3). At location 2, mulching didn’t have any significant effect on plant height (Table 4). The interaction between irrigation and mulching had no significant effect on plant height at both locations (Tables 3 and 4).

#### Leaf area index (LAI).

At location 1, irrigation had a significant effect on LAI. The highest LAI (129.23 ± 14.3) was measured under *I*_*D*_ and the lowest LAI (118.01 ± 14.61) was observed at *I*_*0*_, both were statistically similar with *I*_*C*_ (125.11 ± 23.65) (Table 3). At location 2, irrigation significantly boosted LAI. The maximum LAI (125.85 ± 15.35) was observed under *I*_*D*_, whereas the minimum LAI (86.00 ± 11.98) occurred under *I*_*0*_ (Table 4). Mulching had a significant effect on LAI at location 1, peak LAI (143.13 ± 10.67) was recorded under *M*_*P*_, while the lowest (105.56 ± 5.80) was found in the *M*_*0*_ (Table 3). At location 2, a similar trend was observed. The highest LAI (119.01 ± 23.75) was found under *M*_*P*_, whereas the lowest LAI (94.67 ± 18.37) was recorded in the *M*_*0*_, which was statistically similar with *M*_*S*_ (102.39 ± 20.42) (Table 4). At location 1, the interaction between irrigation and mulching significantly affected leaf area index (LAI). Maximum LAI (153.61 ± 9.66) was measured under *I*_*C*_ *× M*_*P*_, which was statistically similar with *I*_*D*_ *× M*_*P*_ (140.97 ± 3.38). The lowest LAI (101.65 ± 6.03) was observed under *I*_*C*_ *× M*_*0*_ (Table 3). At location 2, the interaction of irrigation and mulching had insignificant effect on LAI (Table 4).

#### Irrigation water use efficiency.

At location 1, irrigation water use efficiency was found to be highest in the conventional irrigation and plastic mulching combination (38.84 kg ha^-1^ mm^-1^), however drip irrigation performed better with plastic and straw mulching setup (Table 5). On the contrary, at location 2 drip irrigation performed better than conventional irrigation in every combination. Plastic mulching with drip irrigation performed the best (65.87 kg ha^-1^ mm^-1^) in terms of water use efficiency (Table 5).

**Table 5:** Comparison of Irrigation water use efficiency of irrigation method to two study locations (kg ha^-1^ mm^-1^).

Treatments	Location 1	Location 2
*I*_*C*_ *× M*_*0*_	2.53	16.93
*I*_*C*_ *× M*_*S*_	6.48	23.64
*I*_*C*_ *× M*_*P*_	38.84	30.06
*I*_*D*_ *× M*_*0*_	4.88	23.13
*I*_*D*_ *× M*_*S*_	25.89	42.3
*I*_*D*_ *× M*_*P*_	16.56	65.87

### Plant stress parameters

#### Hydrogen peroxide (H_2_O_2_).

At location 2, irrigation significantly affected hydrogen peroxide (H_2_O_2_) content in brinjal leaves. Most H_2_O_2_ concentration (119.56 ± 45.83 nmol g^-1^ FW) was determined in *I*_*0*_, whereas the lowest H_2_O_2_ level (108.74 ± 45.05 nmol g^-1^ FW) was determined in *I*_*D*_ (Fig 3). Mulching had a significant effect on hydrogen peroxide (H_2_O_2_) accumulation in plant leaves at location 2 The highest H_2_O_2_ concentration (172.08 ± 11.81 nmol g^-1^ FW) was observed in the *M*_*0*_ and the lowest H_2_O_2_ level (66.32 ± 5.24 nmol g^-1^ FW) was recorded under *M*_*P*_ (Fig 3). The interaction between irrigation and mulching on (H_2_O_2_) content was not significant (Table 6).

**Table 6:** Effect of irrigation and mulching interaction on H_2_O_2_, MDA, CAT, APX and Proline content at location 2.

Treatment	H_2_O_2_ (nmol g^-1^ FW)	MDA (nmol g^-1^ FW)	CAT (EU mg^-1^ protein)	APX (EU mg^-1^ protein)	Proline content (nmol g^-1^ FW)
*I*_*0*_ *× M*_*0*_	175.43 ± 9.28	0.13 ± 0.01	0.6 ± 0.03 b	0.89 ± 0.05 b	0.3 ± 0.01 a
*I*_*0*_ *× M*_*P*_	71.37 ± 1.79	0.11 ± 0.01	0.38 ± 0.01 d	0.13 ± 0.01 ef	0.13 ± 0.01 e
*I*_*0*_ *× M*_*S*_	111.88 ± 7.6	0.12 ± 0.01	0.59 ± 0.03 b	0.21 ± 0.01 cd	0.22 ± 0.01 b
*I*_*C*_ *× M*_*0*_	164.25 ± 12.09	0.12 ± 0.01	0.71 ± 0.03 a	0.86 ± 0.05 b	0.22 ± 0.02 b
*I*_*C*_ *× M*_*P*_	63.56 ± 4.93	0.09 ± 0.01	0.46 ± 0.01 c	0.06 ± 0 f	0.14 ± 0.01 de
*I*_*C*_ *× M*_*S*_	98.41 ± 10.2	0.11 ± 0.01	0.61 ± 0.03 b	0.26 ± 0.02 c	0.2 ± 0.01 b
*I*_*D*_ *× M*_*0*_	176.56 ± 13.67	0.1 ± 0.01	0.63 ± 0.02 b	1.38 ± 0.06 a	0.17 ± 0.01 c
*I*_*D*_ *× M*_*P*_	64.04 ± 4.99	0.09 ± 0.01	0.42 ± 0.03 cd	0.07 ± 0 f	0.12 ± 0.01 e
*I*_*D*_ *× M*_*S*_	90.76 ± 9.65	0.09 ± 0.01	0.46 ± 0.03 c	0.17 ± 0.01 de	0.16 ± 0.01 cd
Sig. level	ns	ns	**	**	**
CV (%)	8.06	7.96	4.87	6.8	4.73

Values are mean ± standard deviation of means; I0: no irrigation (rainfed), *I*_*C*_: conventional furrow irrigation, *I*_*D*_: drip irrigation; M0: no mulching, *M*_*P*_: plastic mulching, *M*_*S*_: straw mulching; and designate significant at 99% and 95% level of significance; NS: not significant: CV: co-efficient of variation; Means within a column followed by the same letter or without a letter are not significantly different at the 5% probability level according to Tukey’s HSD test. ** indicates significance at *p* < 0.01; ns indicates a nonsignificant effect (*p* ≥ 0.05).

**Fig 3:**
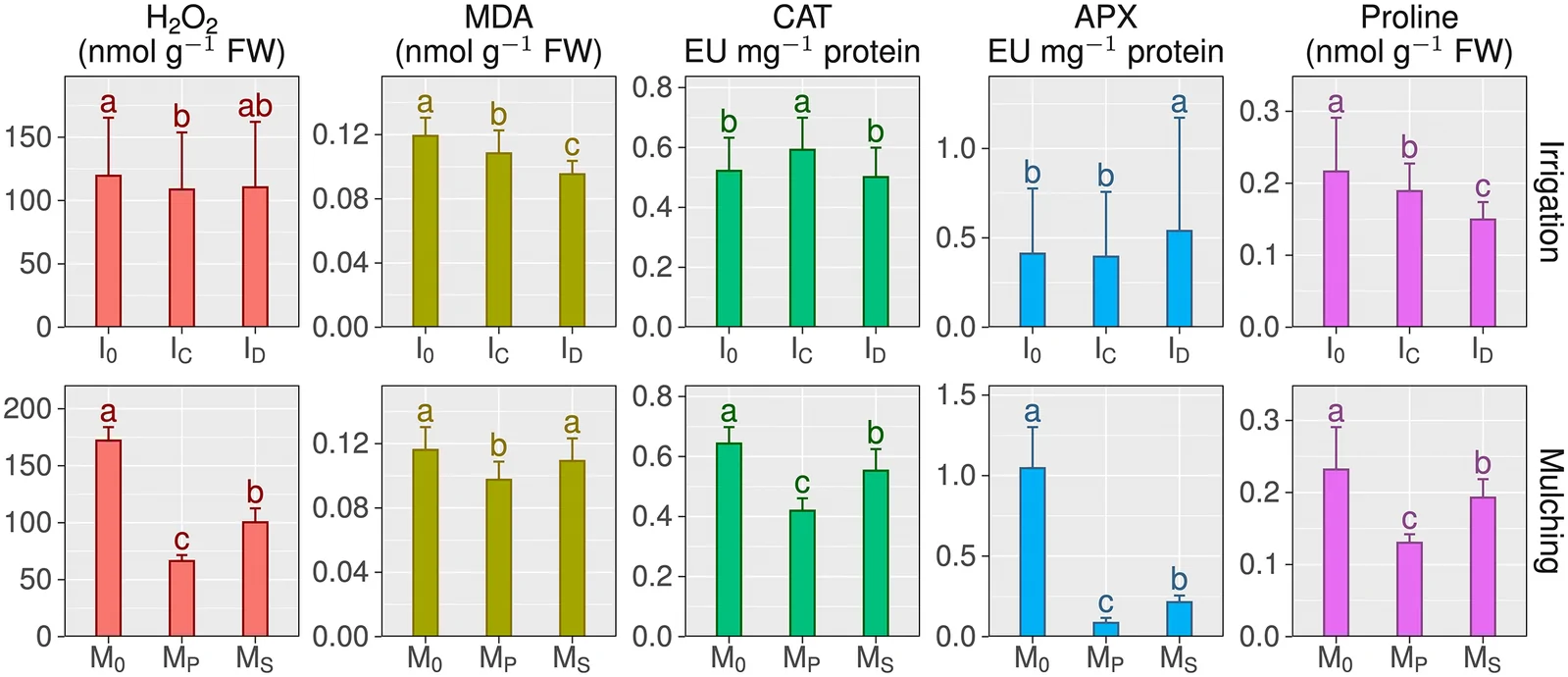
Effect of irrigation and mulching on H_2_0_2_, MDA, CAT, APX and Proline Content. *I*_*0*_: Rainfed, *I*_*C*_: Conventional furrow irrigation and *I*_*D*_: Drip irrigation. M_0_: no mulch, MB: mulching with black plastic and *M*_*S*_: straw mulching; values are mean ± standard deviation; treatment with similar letter or without any letter are statistically significant at P = 0.05.

#### Malondialdehyde (MDA) content.

At location 2, irrigation significantly impacted malondialdehyde (MDA) content in brinjal leaves. The highest MDA concentration (0.12 ± 0.01 nmol g^-1^ FW) was observed in *I*_*0*_ and lowest MDA level (0.1 ± 0.01 nmol g^-1^ FW) was determined in *I*_*C*_ (Fig 3). Mulching had a significantly lowered malondialdehyde (MDA) accumulation in plant leaves at location 2, MDA content peaked (0.12 ± 0.01 nmol g^-1^ FW) in *M*_*0*_*,* which was statistically similar with *M*_*S*_ (0.11 ± 0.01 nmol g^-1^ FW). The lowest MDA concentration (0.1 ± 0.01 nmol g^-1^ FW) was determined in *M*_*P*_ (Fig 3). At location 2, the interaction between irrigation and mulching on MDA content was not significant (Table 6).

#### Catalase (CAT) content.

Irrigation had a significant effect on catalase (CAT) activity at location 2. The highest CAT activity (0.59 ± 0.11 U mg^-1^ protein) was recorded in (*I*_*C*_), whereas the lowest CAT activity (0.5 ± 0.10 EU mg^-1^ protein) was observed in *I*_*D*_, which was statistically similar with *I*_*0*_ (0.52 ± 0.11 EU mg^-1^ protein) (Fig 3). CAT activity was significantly boosted due to mulching at location 2. The highest CAT activity (0.64 ± 0.05 U mg^-1^ protein) was determined in *M*_*0*_, while the lowest CAT activity (0.42 ± 0.04 EU mg^-1^ protein) was observed in *M*_*P*_ treatment (Fig 3). At location 2, *(I × M)* significantly affected catalase (CAT) activity in brinjal leaves. The highest CAT activity (0.71 ± 0.03 EU mg^-1^ protein) was observed under *I*_*C*_ *× M*_*0*_. The lowest CAT activity (0.38 ± 0.01 EU mg^-1^ protein) was observed under *I*_*0*_ *× M*_*P*_, which was statistically similar with *I*_*D*_ *× M*_*P*_ (0.42 ± 0.03, EU mg^-1^ protein) (Table 6).

#### Ascorbate peroxidase (APX) content.

Irrigation had a significant effect on ascorbate peroxidase (APX) activity at location. The highest APX activity (0.54 ± 0.63 U mg^-1^ protein) was measured under *I*_*D*_. The lowest APX activity (0.41 ± 0.36 EU mg^-1^ protein) occurred under *I*_*0*_, which was statistically similar with *I*_*C*_ (0.39 ± 0.3 EU mg^-1^ protein) (Fig 3). Mulching significantly influenced APX activity at location 2. The highest APX activity (1.05 ± 0.26 EU mg^-1^ protein) was estimated under *M*_*0*_, whereas the lowest APX activity (0.09 ± 0.03 EU mg^-1^ protein) was observed *M*_*P*_ (Fig 3). At location 2, the interaction between irrigation and mulching significantly affected ascorbate peroxidase (APX) activity in brinjal leaves. The highest APX activity (1.38 ± 0.06 EU mg^-1^ protein) was found in *I*_*D*_ *× M*_*0*_. The lowest APX activity (0.06 ± 0.00 EU mg^-1^ protein) was observed in *I*_*C*_ *× M*_*P*_ which was statistically similar with *I*_*D*_ *× M*_*P*_ (0.07 ± 0.00 EU mg^-1^ protein) and *I*_*0*_ *× M*_*P*_ (0.13 ± 0.01 EU mg^-1^ protein) (Table 6).

#### Proline content.

Significant reduction in proline content was observed due to sufficient irrigation at location 2. Proline content peaked (0.22 ± 0.07 nmol g-^1^ FW) in *I*_*0*_, whereas it dipped at *I*_*D*_ (0.15 ± 0.02 nmol g-^1^ FW) (Fig 3). Mulching lowered the proline content significantly. Most amount of proline content (0.23 ± 0.06 nmol g-^1^ FW) was recorded under the *M*_*0*_, and the lowest amount (0.13 ± 0.01 nmol g-^1^ FW) was observed under *M*_*P*_ (Fig 3). The interaction *I × M* had a significant effect on proline content in leaves. The highest proline content was found (0.3 ± 0.01 µmol g-^1^ FW) in *I*_*0*_ *× M*_*0*_ and the lowest (0.12 ± 0.01 nmol g-^1^ FW) was found in *I*_*D*_ *× M*_*P*_, which was statistically similar with *I*_*0*_ *× M*_*P*_ (0.13 ± 0.01 nmol g-^1^ FW) and *I*_*C*_ *× M*_*P*_ (0.14 ± 0.01 nmol g-^1^ FW) (Table 6).

### Field conditions

#### Soil moisture content.

Soil moisture content was significantly influenced by irrigation at both locations. Maximum moisture content was measured in *I*_*C*_ (27.62 ± 2.49%) at location 1, which was statistically similar with *I*_*D*_ (27.36 ± 4.38) (Fig 4). Similarly, highest moisture content (24.01 ± 4.41) was found in *I*_*C*_ at location 2, which was statistically similar *I*_*D*_ (22.11 ± 4.66)*.* The lowest amount was found under *I*_*0*_ at both locations, (22.27 ± 3.63%) and (19.13 ± 2.62%) respectively (Fig 4). At location 1, mulching significantly improved soil moisture content. The highest soil moisture (29.57 ± 2.85%) was measured in *M*_*P*_, while the lowest value (22.09 ± 3.02%) was observed in the *M*_*0*_ treatment (Fig 4).

**Fig 4:**
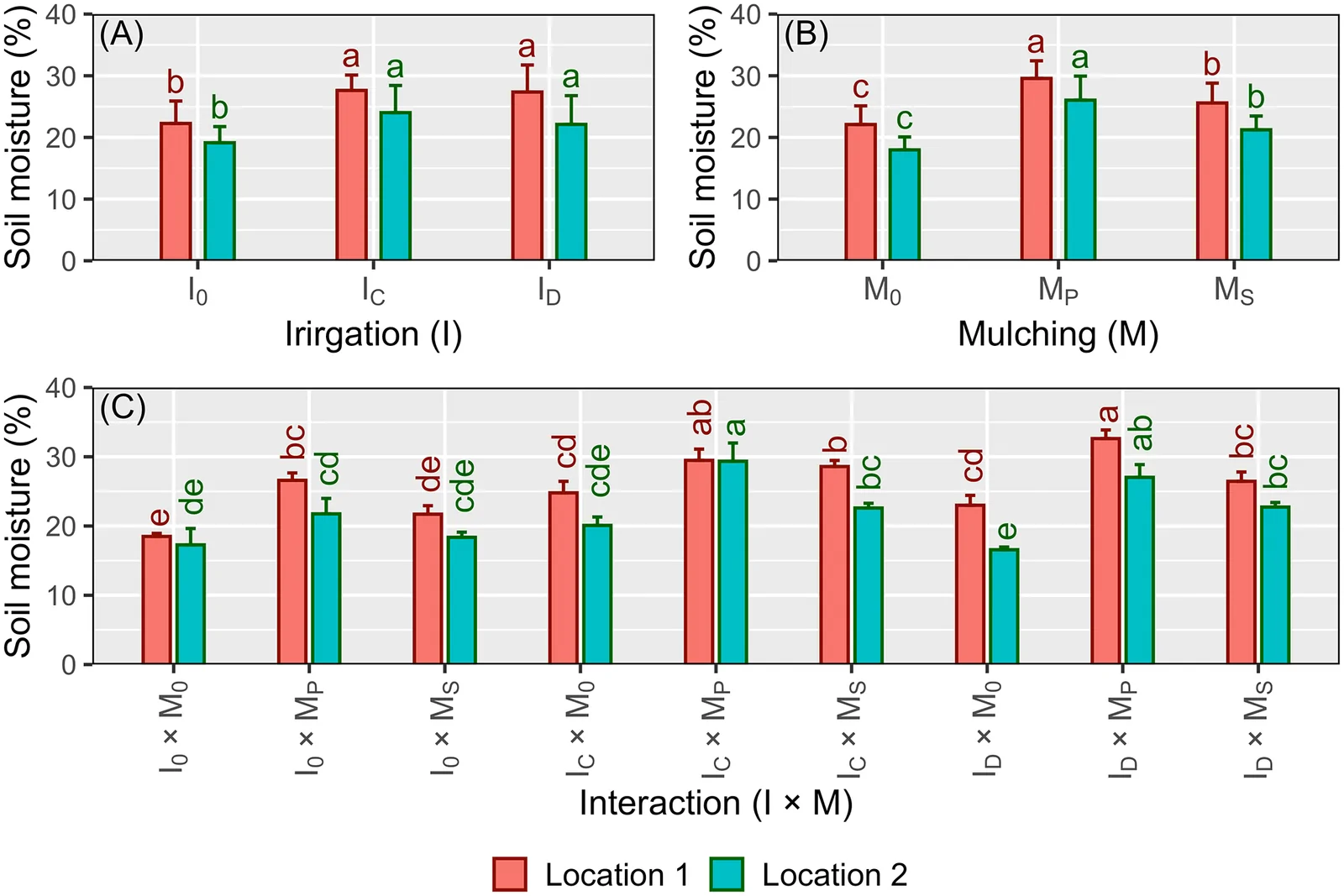
Effect of irrigation and mulching on soil moisture (%) content. *I*_*0*_: Rainfed, *I*_*C*_: Conventional furrow irrigation and *I*_*D*_: Drip irrigation. M_0_: no mulch, MB: mulching with black plastic and *M*_*S*_: straw mulching; values are mean ± standard deviation; treatment with similar letter or without any letter are statistically significant at P = 0.05.

At location 2, a similar pattern of increasing soil moisture due to mulching was observed. *M*_*P*_ maintained the highest soil moisture (26.05 ± 3.90%) and *M*_*0*_ had the lowest soil moisture (17.98 ± 2.10%) (Fig 4). At location 1, interaction of irrigation and mulching significantly affected soil moisture content. Peak soil moisture content (32.63 ± 1.24%) was measured in *I*_*D*_ *× M*_*P*_*,* which was statistically similar with *I*_*C*_ *× M*_*P*_ (29.48 ± 1.62%). The lowest soil moisture (18.49 ± 0.45%) was found under *I*_*0*_ *× M*_*0*_, which was statistically similar with *I*_*0*_ *× M*_*S*_ (21.71 ± 1.23) (Fig 4). At location 2, a similar trend was observed. The highest soil moisture (29.35 ± 2.63%) was observed with *I*_*C*_ *× M*_*P*_, statistically similar with *I*_*D*_ *× M*_*P*_ (27.03 ± 1.84%). The lowest value (16.58 ± 0.39%) occurred under *I*_*D*_ *× M*_*0*_, which was statistically similar with *I*_*C*_ *× M*_*0*_, *I*_*0*_ *× M*_*0*_ and *I*_*0*_ *× M*_*S*_ (Fig 4).

#### Soil temperature.

Irrigation treatment did not affect the soil temperature significantly at both locations (Fig 5). At location 1, mulching significantly increased soil temperature. The highest temperature (26.96 ± 0.67 °C) was recorded in *M*_*P*_, which was statistically similar to the *M*_*0*_ treatment (26.81 ± 0.78 °C). The lowest soil temperature (25.11 ± 0.67 °C) was found under *M*_*S*_ (Fig 5). At location 2, soil temperature was significantly affected by mulching. *M*_*P*_ maintained maximum temperature (26.74 ± 0.81 °C) and *M*_*S*_ showed the lowest value at (25.13 ± 0.68 °C), both were statistically similar with *M*_*0*_ (25.85 ± 1.32) (Fig 5). The interaction between irrigation and mulching had no significant impact on soil temperature at both locations (Fig 5).

**Fig 5:**
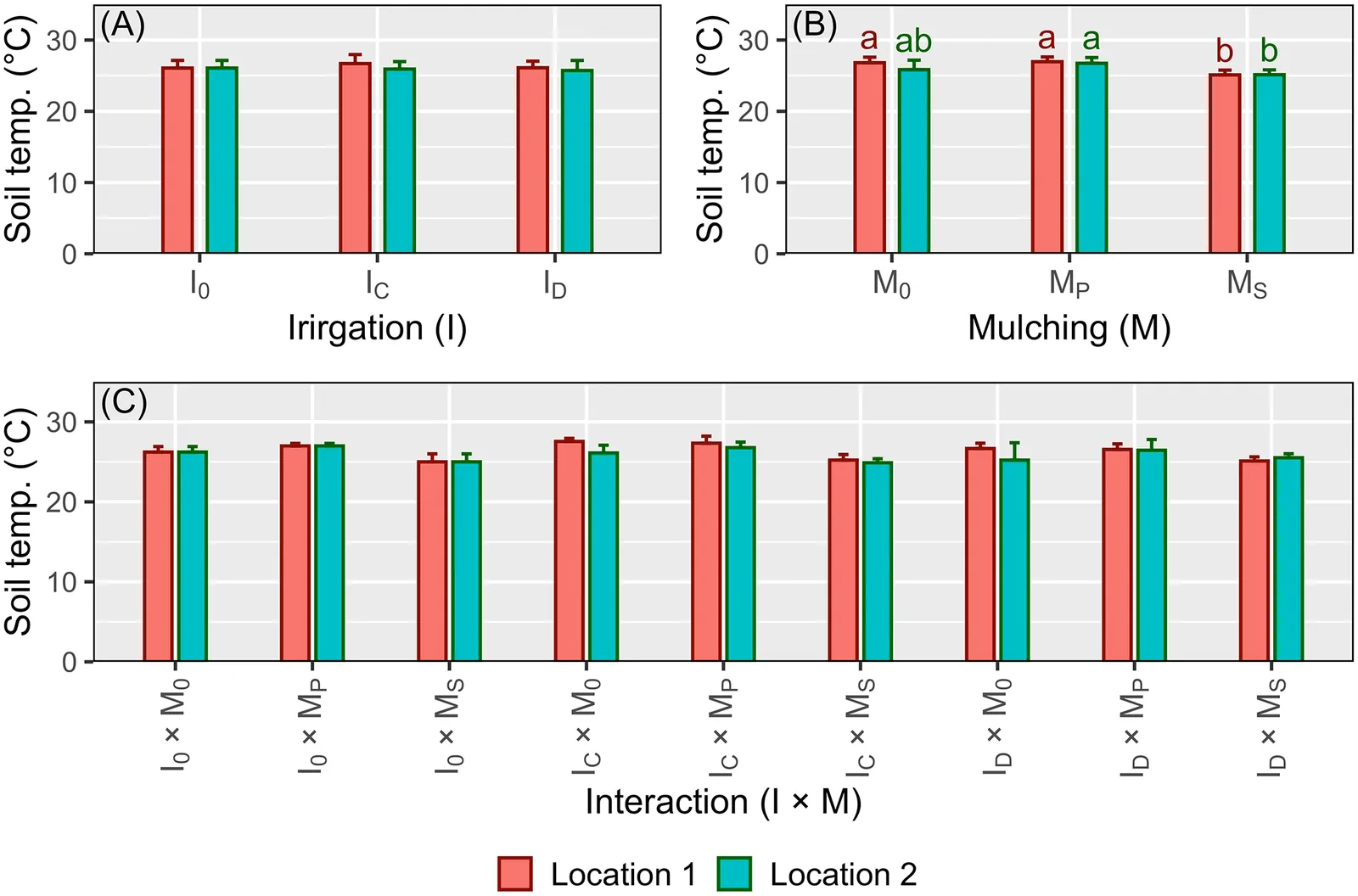
Effect of irrigation and mulching on soil temperature. *I*_*0*_: Rainfed, *I*_*C*_: Conventional furrow irrigation and *I*_*D*_: Drip irrigation. M_0_: no mulch, MB: mulching with black plastic and *M*_*S*_: straw mulching; values are mean ± standard deviation; treatment with similar letter or without any letter are statistically significant at P = 0.05.

#### Weed dry weight.

At location 1, weed dry weight was significantly influenced by irrigation. Highest weed dry weight (10.58 ± 8.28 g m^-2^) was determined in *I*_*C*_ and lowest (7.6 ± 4.65 g m^-2^) was measured in *I*_*D*_, both were statistically similar with *I*_*0*_ (8.54 ± 5.6) (Fig 6). At location 2, the effect of irrigation was not significant (Fig 6). Mulching significantly suppressed weed growth at both locations, following similar pattern. At location 1, the lowest weed biomass (1.48 ± 0.27 g m^-2^) was recorded in *M*_*P*_, whereas the highest value (14.57 ± 4.69 g m^-2^) was observed in the *M*_*0*_ treatment (Fig 6). At location 2, *M*_*P*_ resulted in the lowest weed dry weight (0.99 ± 0.45 g m^-2^) and *M*_*0*_ treatment produced the highest weed biomass (12.88 ± 3.00 g m^-2^) (Fig 6). At location 1, the interaction between irrigation and mulching had a significant influence on weed dry weight. The highest weed dry weight (19.19 ± 4.96 g m^-2^) was measured in *I*_*C*_ *× M*_*0*_, which was statistically similar with *I*_*0*_ *× M*_*0*_ (13.46 ± 3.01 g m^-2^). In contrast, the lowest weed dry weight (1.31 ± 0.09 g m^-2^) was observed under *I*_*C*_ *× M*_*P*_, which was statistically similar with *I*_*0*_ *× M*_*P*_, (1.61 ± 0.27 g m^-2^) and *I*_*D*_ *× M*_*P*_ (1.53 ± 0.38 g m^-2^) (Fig 6). At location 2, the interaction effect on weed dry weight was not significant (Fig 6).

**Fig 6:**
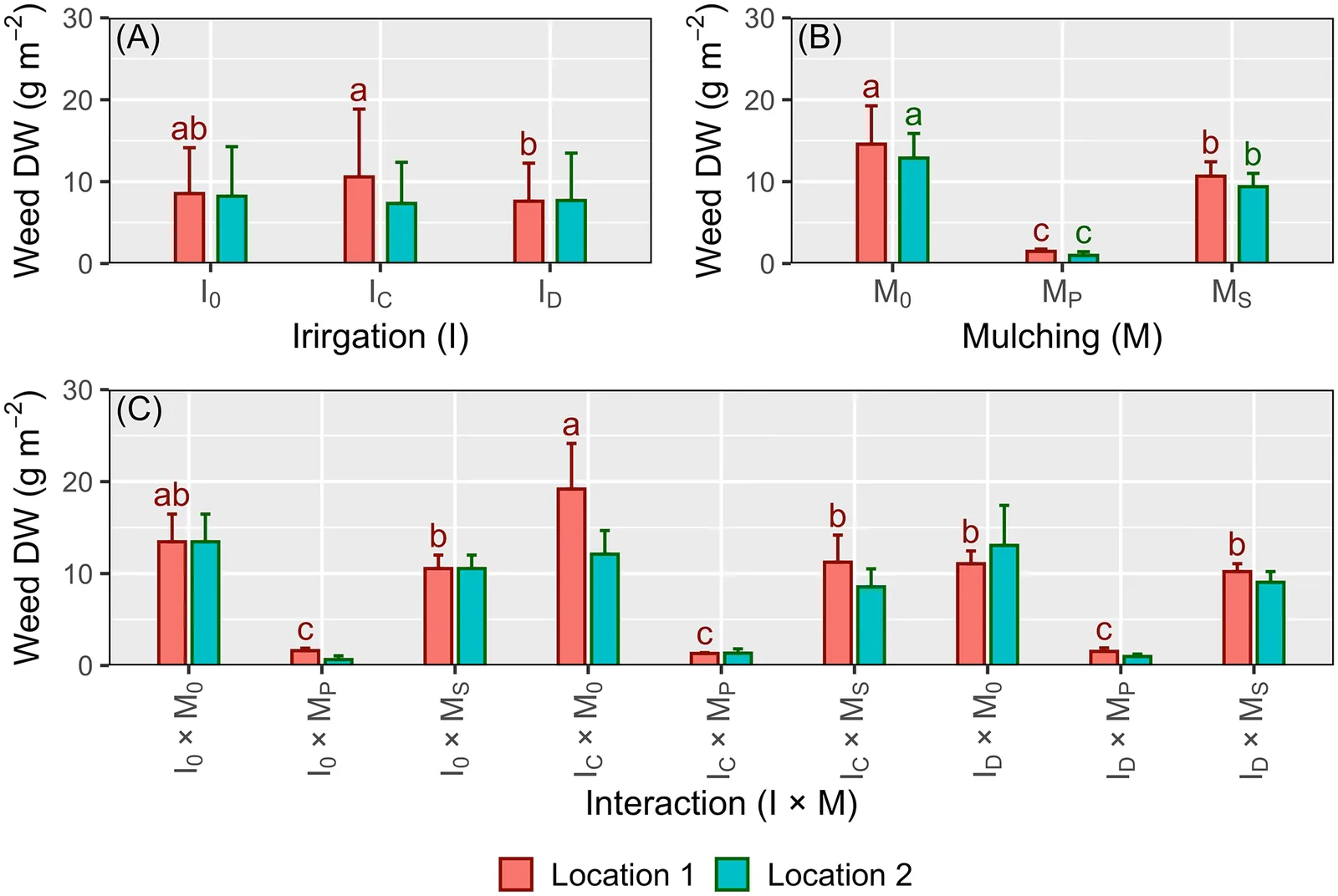
Effect of irrigation and mulching on weed dry weight. *I0*: Rainfed, *I*_*C*_: Conventional furrow irrigation and *I*_*D*_: Drip irrigation. M0: no mulch, MB: mulching with black plastic and *M*_*S*_: straw mulching; values are mean ± standard deviation; treatment with similar letter or without any letter are statistically significant at P = 0.05.

### Brinjal yield

At location 1, brinjal fruit yield was insignificantly affected by irrigation (Fig 7). At location 2, the opposite trend was observed; irrigation significantly increased yield compared to control. The highest yield (21.88 ± 9.4) t ha^-1^ was obtained in *I*_*D*_ and lowest yield (10.1 ± 3.49) tha^-1^ was obtained under *I*_*0*_ (Fig 7). At location 1, mulching had a significant influence on fruit yield. The highest yield (16.71 ± 6.53 t ha^-1^) was obtained under *M*_*P*_, while the lowest yield (2.98 ± 2.43 t ha^-1^) was recorded in the *M*_*0*_ treatment (Fig 7). At location 2, a similar trend was observed yield. *M*_*P*_ had the highest yield (24.02 ± 8.63 t ha^-1^), whereas the lowest yield (9.34 ± 2.75 t ha^-1^) occurred in the *M*_*0*_ treatment (Fig 7). At Location 1, *I*_*C*_ *× M*_*P*_ produced the highest numerical fruit yield (23.18 ± 6.62 t ha^-1^), but it was not significantly different from *I*_*D*_ *× M*_*S*_ (12.93 ± 4.53 t ha^-1^; pairwise *p* = 0.079) and *I*_*0*_ *× M*_*P*_ (15.61 ± 2.96 t ha^-1^; pairwise *p* = 0.333), likely due to the relatively high experimental variability (CV = 20.86%). The lowest yield (1.52 ± 0.76 t ha^-1^) was recorded under *I*_*C*_ *× M*_*0*_ (Fig 7). At location 2, the interaction also showed a significant effect on brinjal yield. The maximum yield (32.93 ± 1.62 t ha^-1^) was obtained under *I*_*D*_ *× M*_*P*_, whereas the lowest yield (6.31 ± 2.22 t ha^-1^) was observed under *I*_*0*_ *× M*_*0*_.

**Fig 7:**
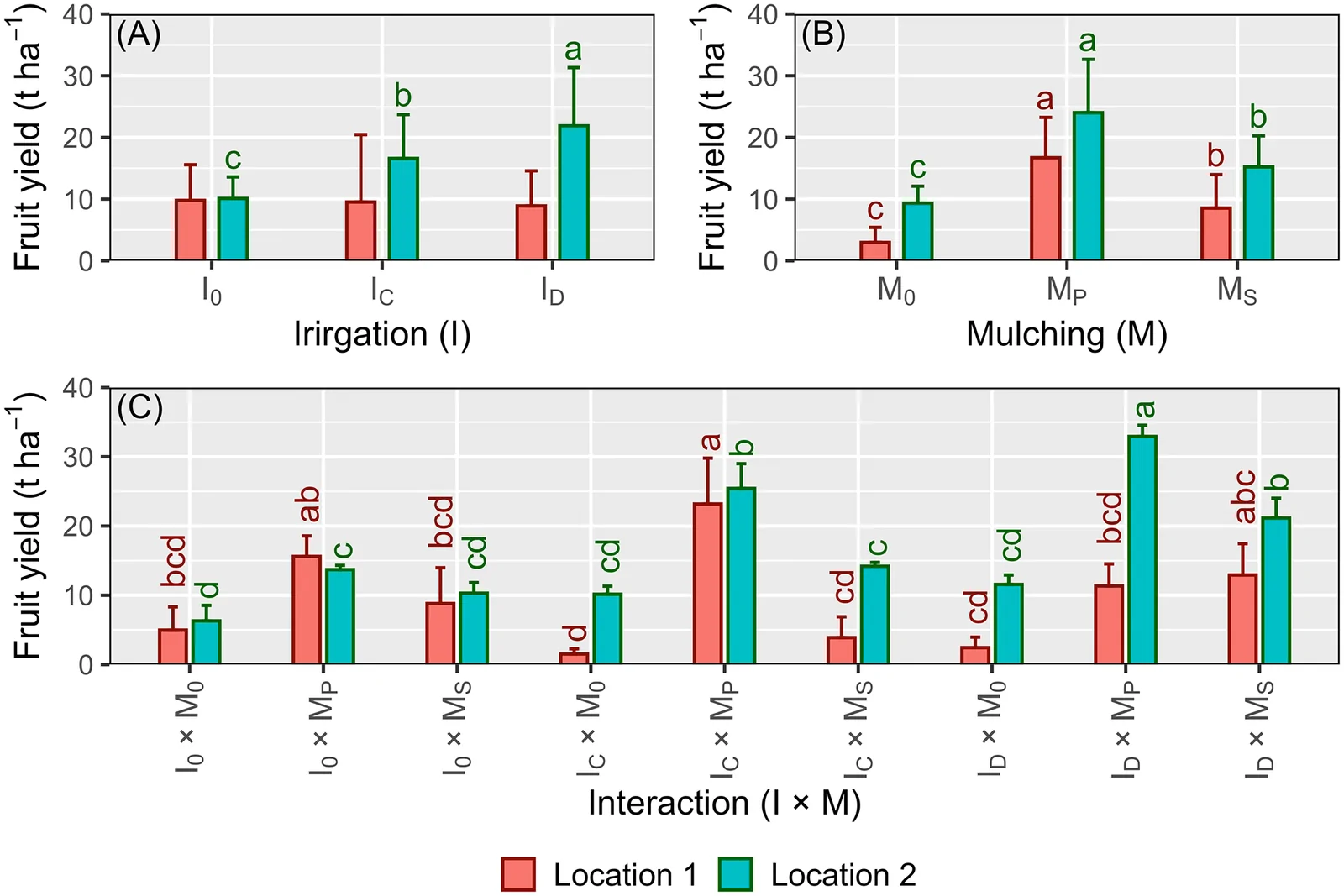
Effect of irrigation and mulching on fruit yield of brinjal. *I0*: Rainfed, *I*_*C*_: Conventional furrow irrigation and *I*_*D*_: Drip irrigation. M0: no mulch, MB: mulching with black plastic and *M*_*S*_: straw mulching; values are mean ± standard deviation; treatment with similar letter or without any letter are statistically significant at P = 0.05.

### Modeling brinjal yield

A linear mixed model (LMM) (estimated using REML and ‘nloptwrap’ optimizer) to predict brinjal fruit yield with irrigation, mulching and location (field). The model included replication as random effect, and irrigation, mulching and study location were considered the fixed effects. The model’s total explanatory power is substantial (conditional *R*^2^ = 0.88) and the part related to the fixed effects alone (marginal *R*^2^) is of 0.88. The model’s intercept, corresponding to irrigation = *I*_*0*_, mulch = *M*_*0*_ and location = location 1 (Agron), is at 4.98 (95% CI [1.39, 8.56], t (34) = 2.82, p = 0.008). The model had no problem related to heteroscedasticity and overdispersion of residuals as diagnosed by the ‘DHARMa’ package (S1 Fig).

Model estimates of brinjal yield due to different treatment levels and their combinations are presented in Table 7.

**Table 7:** Fixed effect estimates as derived from the linear mixed-effect model (LMM).

	Estimate	Std. error	df	t value	p-value	Sig. level	CI (2.5% − 95%)
Intercept	4.98	1.76	35.96	2.82	0.008	**	2.1 to 7.85
*I*_*C*_	−3.46	2.48	34.00	−1.39	0.173	ns	−7.51 to 0.59
*I*_*D*_	−2.54	2.48	34.00	−1.02	0.314	ns	−6.59 to 1.51
*M*_*P*_	10.63	2.48	34.00	4.28	0.000	***	6.58 to 14.68
*M*_*S*_	3.83	2.48	34.00	1.54	0.132	ns	−0.22 to 7.88
*Loc 2*	1.34	2.48	34.00	0.54	0.594	ns	−2.71 to 5.39
*I*_*C*_ *× M*_*P*_	11.03	3.51	34.00	3.14	0.003	**	5.3 to 16.76
*I*_*D*_ *× M*_*P*_	−1.73	3.51	34.00	−0.49	0.626	ns	−7.46 to 4
*I*_*C*_ *× M*_*S*_	−1.46	3.51	34.00	−0.42	0.679	ns	−7.19 to 4.26
*I*_*D*_ *× M*_*S*_	6.66	3.51	34.00	1.90	0.067	ns	0.93 to 12.38
*I*_*C*_ *× Loc2*	7.30	3.51	34.00	2.08	0.045	*	1.57 to 13.03
*I*_*D*_ *× Loc2*	7.79	3.51	34.00	2.22	0.033	*	2.06 to 13.51
*M*_*P*_ *× Loc2*	−3.25	3.51	34.00	−0.92	0.362	ns	−8.97 to 2.48
*M*_*S*_ *× Loc2*	0.15	3.51	34.00	0.04	0.965	ns	−5.57 to 5.88
*I*_*C*_ *× M*_*P*_ *× Loc2*	−3.14	4.97	34.00	−0.63	0.531	ns	−11.24 to 4.96
*I*_*D*_ *× M*_*P*_ *× Loc2*	15.71	4.97	34.00	3.16	0.003	**	7.61 to 23.81
*I*_*C*_ *× M*_*S*_ *× Loc2*	1.51	4.97	34.00	0.31	0.762	ns	−6.59 to 9.61
*I*_*D*_ *× M*_*S*_ *× Loc2*	−1.06	4.97	34.00	−0.21	0.833	ns	−9.16 to 7.04

*, **, and *** indicate significance at p < 0.05, p < 0.01, and p < 0.001, respectively; ns indicates a nonsignificant effect (p ≥ 0.05).

The linear mixed model revealed a highly significant main effect of plastic mulching (*p* < 0.001), which increased yield by approximately 10.6 t ha^-1^ relative to the no-mulch control. The effects of irrigation levels alone were not significant, but irrigation interacted significantly with both mulching and location. In particular, under irrigation level *I*_*C*_, the use of plastic mulch substantially improved yield (p = 0.003). The effects of irrigation levels *I*_*C*_ and *I*_*D*_ also varied significantly between locations (*p* = 0.045 and 0.033, respectively), indicating that irrigation response was location dependent.

A significant three-way interaction (*I*_*D*_ *× M*_*P*_ *× Loc2*; p = 0.003) further suggested that the yield advantage of plastic mulch under irrigation *I*_*D*_ was particularly strong (or different) at Location 2. Other two- and three-way interactions were not statistically significant, implying that the main beneficial effect of plastic mulch was generally consistent across irrigation and location conditions, except for the noted cases. Therefore, the model suggests that only the *I*_*D*_ *× M*_*P*_ *× Loc2* interaction is truly significant, suggesting that the benefit of plastic mulch under irrigation *I*_*D*_ depends on the location.

## Discussion

This study shows that mulching treatments, especially the plastic mulch, favored the performance of brinjal across soil types. However, brinjal’s performance towards irrigation regimes, particularly that of drip irrigation was location specific and it was strongly dependent on the water holding capacity of the soil. At both locations, plastic mulch conserved a higher amount of soil moisture than no mulching and straw mulching treatments and thus increased the water use efficiency of the crop. It also improved the soil microclimate (soil temperature) and suppressed weed and thus increased the growth of brinjal plants (SPAD value, leaf area index). Since plastic mulch preserved optimum soil moisture, the plants suffered less from oxidative stress. These positive effects of plastic mulch were translated into higher fruit yield compared to no mulching and straw mulching. The responses of brinjal to irrigation treatments, on the other hand, was different at the study sites. Location 2 was characterized by higher organic matter content and silt loam texture, where growth and brinjal yield was increased substantially by drip irrigation compared to no irrigation or the conventional furrow irrigation. However, at location 1, where the soil is coarser and had lower water retention capacity, drip irrigation did not show any yield advantages over the other irrigation treatments.

SPAD meters are routinely used in field conditions to measure greenness (SPAD value) of leaves. Though there are some limitations of considering the SPAD value as an alternative approach of understanding leaf chlorophyll content (LCC) [46,47], it is being widely used to predict the photosynthetic activity of the leaf and high values are translated to healthier growth of the plant [48]. Mulching significantly improved SPAD value at both locations compare to the zero-mulching plot. Mulches preserve soil moisture content [23] and provide thermal insulation [49] which facilitates the root growth of the plant which ultimately leads to increased leaf number and photosynthetic area [50]. In contrast to the previous findings, SPAD value was found to be significantly higher in the non-irrigated plot compared to drip irrigation and conventional furrow irrigation in our study. A higher SPAD value under non-irrigated treatment compared to drip irrigation may be a sign of increased relative chlorophyll concentration per unit leaf area. However, leaf structural and physiological characteristics may also influence SPAD readings. Because leaf thickness, specific leaf area and extracted chlorophyll concentration were not measured, the mechanism underlying this response could not be determined. Therefore, the increased SPAD value should not be taken as direct evidence of thicker leaves or improved physiological performance. Abd El-Mageed, Ibrahim [51] and Nemeskéri, Molnár [52] reported similar results where they found that SPAD value was higher in moisture stress condition compared to well irrigated plot in potato and snap bean, respectively. Irrigation regime alone and its interaction with mulching though showed positive effect on SPAD value at location 1, it was not affected at location 2. It was reported that plants may response differently to irrigation under different soil types and management [53]. The relatively low SPAD value in *I*_*C*_ *× M*_*S*_ treatment at location 1 may be attributed to plot-specific variability rather than a treatment response. Leaf greenness may have been reduced in this treatment combination due to localized factors such as temporary nutrient immobilization, incidence of pest or disease, or measurement variability.

At both locations, drip irrigation significantly improved plant height compared to conventional furrow irrigation and no irrigation. In drip irrigation systems, water loss is minimal due to evaporation and water is delivered in small amounts in frequent intervals, which ultimately maintains the constant supply of water needed by plant increasing plant height [54], and the water retention and plant growth is even higher if a drip irrigated crop is mulched with plastic film [27,55]. For the same reason, leaf area index (LAI) of brinjal had significant effect from both irrigation regime and mulching management and was favored by drip irrigation and plastic mulch irrespective of soil type. LAI was found to be higher in plastic mulch, and the effect was significant. *M*_*P*_ yielded ~26% higher LAI compare to control at both locations. Ye, Li [56] reported higher LAI in wheat under mulched condition.

At location 1, performance of drip irrigation was not consistent and in contrast, location 2 had better water use efficiency in terms of drip irrigation in comparison to conventional furrow irrigation. At both locations, plastic mulching in combination with different irrigation system performed well and maintained higher irrigation water use efficiency (IWUE). Water use efficiency was found to be 14% higher in drip irrigation than furrow irrigation in cotton [57], and up to 50% in brinjal [58,59]. However, similar response was not observed at Location 1. This divergence may be explained by site-specific soil and management conditions. Location 1 had coarser soil, lower organic matter content, and lower water-holding capacity, which may have limited the effectiveness of the applied drip-irrigation schedule. In coarse-textured soils, water applied through drip emitters can drain rapidly and may produce a relatively restricted wetted zone if emitter discharge, spacing, and irrigation frequency are not well matched with soil hydraulic properties and crop water demand. Under these conditions, conventional furrow irrigation may temporarily wet a larger portion of the root zone, resulting in better crop performance and IWUE than drip irrigation.

Soil temperature in this study was not found to be significantly affected by the irrigation regimes. Previous studies have reported that plots with drip irrigation had lower soil temperature compared to no irrigation plots or the same with border irrigation [60]. Soil moisture content gradually decreases over time after irrigation. In this study, the soil temperature data was recorded at a certain interval during when the temperature changes due to soil moisture may have been undetected.

Soil moisture content and irrigation regime determine weed composition and dry mass production [61,62]. At location 1, the highest weed dry mass was recorded in conventional irrigation and the lowest in drip irrigation. Weeds grow vigorously at high soil moisture condition [61]. The scarce growth of weeds under drip irrigation systems can be explained by the localized application of water. Under drip irrigation, only the root zone is kept wet while the rest of the field is not irrigated. However, no irrigation gave a similar weed dry weight to the conventional furrow irrigation, which does not match with previous research findings. The inherent soil seed bank may have some role in this unusual weed infestation. At location 2, weed dry mass did not differ for irrigation regimes. A higher moisture holding capacity may have provided equal advantages to the weeds for all irrigation treatments.

While abiotic stress causes excessive generation of reactive oxygen species (ROS) (e.g., H_2_O_2_ in this study) plant tissue [63–65], high concertation of malondialdehyde (MDA) indicates oxidative damage of plant cell walls [66]. H_2_O_2_ and MDA concentrations in the leaf tissues of brinjal, in this study, were in consistent with the soil microclimate (soil moisture content and soil temperature) mediated by soil characteristics of the locations, mulching and irrigation management and their interactions. H_2_O_2_ was found to be higher in the control (non-irrigation) plot compared to drip and conventional irrigation due to higher oxidative stress. H_2_O_2_ values at location 1 suggest that drip irrigation was not comparable to conventional furrow irrigation and the plants could not evade drought stress because the site has coarser soil and lower water holding capacity compared to location 2. In addition, there was no rainfall until the later stage of the crop (Fig 2) to sustain the plants under no or drip irrigation at location 1. Excessive generation and accumulation of antioxidative enzymes (e.g., catalase (CAT) and ascorbate peroxidase (APX)) and non-enzymatic scavengers (e.g., proline) are triggered by ROS and their concentration indicates the level of abiotic stress the plants are suffering [67,68]. In connection with the tissue concentrations of H_2_O_2_ and MDA, the scavenging mechanism of brinjal plants were likely to be activated and the concentrations of CAT, APX and proline showed the similar trend for different mulching and irrigation managements as found for ROS. Location-specific trends for these biochemical markers also indicated the harsh condition at location 1. Overall, mulching improved the soil moisture content and higher soil moisture content was measured under plastic mulching which support previous research findings [25,50].

Field conditions (soil moisture, temperature, weed infestation), growth and canopy development of the plants (SPAD value, LAI, plant height) and biochemical stress markers in plant tissue varied due to irrigation regimes and mulching managements, and their effects were manifested in brinjal fruit yield of the respective plots. However, like other studied parameters, brinjal responded differently for the study locations. Drip irrigation increased ~117% and ~32% fruit yield compared to no irrigation and conventional furrow irrigation, respectively at location 2. Similar result can be found; drip irrigation reduces moisture stress which ensures better flowering and less pre- maturing flowers and fruit drops, regular application of water ensure nutrient absorption and reduces loss of nutrients through leaching and evapotranspiration, which leads to improve yield [69]. The fruit yield obtained from location 1 under drip irrigation was having a high standard deviation among the replicate plots. This may be related to the heterogeneous and coarse textured soil at this site, with relatively low organic matter, nutrient content and water retention capacity. Under these conditions, differences in soil-water retention and distribution within the plot, may lead to uneven crop establishment, fruit maturation and yield across plots. The small number of replications may have increased the effect of individual plot responses on the estimated standard deviation. Hence, the average yield response to drip irrigation at Location 1 should be interpreted with caution, especially as it was not statistically different from the conventional furrow irrigated and rainfed treatments.

There were conspicuous yield differences for mulching treatments at both locations, where plastic mulch had the highest yield followed by straw mulching and no mulching, respectively. Plastic mulch provided a good microclimate for the rootzone and retained much moisture which boosted brinjal yield [29, 70]. Though drip irrigation coupled with plastic mulching performed the best at location 2, it was different at location 1. However, each soil texture was represented by only one field, location and soil type were not truly replicated. Therefore, location should be considered a proxy for the contrasting soil conditions rather than a replicated soil-type treatment. Due to the coarser soil, low organic matter content and water holding capacity, drip irrigation could not provide the plant with enough soil moisture in the root zone. Conventional furrow irrigation with plastic mulch produced the highest yield at location 1.

Plastic mulch had a significant positive effect on fruit yield, with an estimated yield increase of approximately 10.6 t ha^−1^ with respect to no mulch, which was corroborated by the linear mixed model. In addition, the significant irrigation × mulch × location interaction showed that the responses to irrigation were different in the different locations and were influenced by the mulch treatment. Thus, the efficacy of irrigation practices seemed to be influenced by the field conditions at a specific location and the associated mulch-management package.

One major limitation of this study is that biochemical stress markers were only measured at Location 2 due to logistic constraints. Hence the biochemical responses recorded in this study are location specific and cannot be used to compare the differences in the plant stress physiology between the two locations. In particular, the influence of different soil conditions on oxidative stress and antioxidant responses could not be assessed, as similar measurements were not available for Location 1. Thus, the biochemical results should be considered as supporting evidence of treatment responses at Location 2, and not as evidence for location- or soil-specific physiological mechanisms. Future studies should measure the same biochemical and plant-water-status variables at all experimental sites and include replicated fields within each soil class to allow for valid comparisons of physiological responses across environments.

## Supporting information

S1 Fig Diagnosis plots (a, b, and c) for the selected model prepared by the ‘DHARMa’ package of `R’ [43]. (TIFF)

S1 Data Replicated field-trial data from both study sites used for analysis of variance (ANOVA) and linear mixed-effects modeling (LMM). (XLSX)

S1 File R script used for analysis of variance (ANOVA), linear mixed-effects modeling (LMM), and generation of the corresponding results and figures. (R)
