# Supplementary material for: Plastic mulching consistently improves brinjal performance, but drip irrigation benefits are soil dependent: A two-location field study
Source: PLoS One. 2026 Oct 27;21(10):e0360413. doi: 10.1371/journal.pone.0360413 (PMC13712550; doi:10.1371/journal.pone.0360413)
Supplement: Supplementary file 1 [file pone.0360413.s004.docx]

**Professional Academic Peer Review**

**Manuscript: PONE-D-26-17291**

**Title: "Responses of brinjal to irrigation and mulching treatments depends on soil characteristics"**

**Overall Assessment**

This manuscript presents a field study examining the interactive effects of irrigation regimes and mulching practices on brinjal (eggplant) growth, yield, and physiological responses across two contrasting soil types in Bangladesh. The research addresses an important topic in water-scarce agriculture. However, the manuscript contains **significant methodological flaws, data inconsistencies, and presentation errors** that undermine its scientific rigor and clarity. **Major revisions are required before publication can be considered.**

**Major Critical Comments**

**1. Title**

| **Comments** | **Suggestion** |
| --- | --- |
| Grammatical error: "depends" should be "depend" (subject-verb agreement with plural "responses") | Revise to: **"Responses of brinjal to irrigation and mulching treatments depend on soil characteristics"** |
| Title is descriptive but lacks specificity about the key finding | Consider: **"Plastic mulching consistently improves brinjal performance, but drip irrigation benefits are soil-dependent: A two-location field study"** |

**2. Abstract**

| **Comments** | **Suggestion** |
| --- | --- |
| **Critical contradiction**: Line 15 reports drip irrigation yield at Location 1 as 8.9 ± 5.68 t ha⁻¹ (SD = 64% of mean!), yet Line 19 shows 23.18 ± 6.62 t ha⁻¹ under IC×MP. The enormous SD suggests extreme variability or measurement errors | Report coefficients of variation and explain why drip irrigation performed so poorly at Location 1; consider whether the drip system was appropriately designed for sandy soil |
| Line 16 states drip irrigation at Location 1 was "statistically similar to IC and IO" - with such high variance, this conclusion is statistically weak; report actual p-values or confidence intervals | Replace vague "statistically similar" with precise statistical metrics |
| The abstract claims "plastic mulching consistently enhanced brinjal performance" but Line 23 shows Model estimated +10.6 t ha⁻¹ - this needs confidence intervals | Report 95% CI around this estimate |
| Missing key information: duration of study, actual irrigation amounts applied, and explanation of why Location 1 had *lower* yield under drip than rainfed? (8.9 vs not specified for IO) | Add comparative rainfed yield values |

**3. Introduction**

| **Comments** | **Suggestion** |
| --- | --- |
| **Line 48**: "Brinal" typo - should be "Brinjal" | Correct throughout manuscript |
| Lines 29-31: General statements about global water scarcity lack specific citations to brinjal production contexts | Add region-specific statistics for brinjal water requirements in South Asia |
| Lines 53-56: Claim that drip irrigation "increases water productivity and yield compared to conventional furrow" is overly simplistic given your own findings (Location 1 showed no benefit) | Acknowledge early that soil type moderates this response |
| Lines 75-78: The research gap is framed as "performance in contrasting soil types not well documented" - but your own methods lack replication across soil types (only 2 locations, not a gradient) | Reframe as "preliminary comparison between two distinct soil textures" |
| Missing: Justification for the specific irrigation levels (why 20-day intervals for conventional irrigation?) | Add rationale based on local practice or preliminary studies |

**4. Materials and Methods**

| **Comments** | **Suggestion** |
| --- | --- |
| **Critical flaw - Section 2.1, Table 1**: Location 1 is described as "sandy loam" with particle density 2.79 g cc⁻¹, Location 2 as "silt loam" with 2.63 g cc⁻¹ - but **no replication of locations** (each soil type has only ONE field). This confounds soil type with other site-specific factors (history, management, microclimate) | Acknowledge this as a severe limitation in the Discussion; state clearly that "location" is a proxy for soil type, not a true treatment |
| **Line 110**: "Location 2 has a silt loam soil which means better water holding capacity compared to the sandy loam of location 2" - **This is a typo** (should be "location 1") | Correct: "compared to the sandy loam of location 1" |
| **Section 2.3**: Irrigation amounts are inadequately described. "55 mL of water was applied per minute per plant" -这是什么意思? This does not translate to mm without knowing plant spacing and emitter configuration | Provide: emitter flow rate (L h⁻¹), number of emitters per plant, irrigation duration and frequency, total applied water (mm) for each treatment |
| **Line 140**: "Irrigation was given regularly automatically with drip irrigation system" - vague; specify controller settings, pressure, and verification method | Add technical specifications of the drip system (manufacturer, emitter spacing, operating pressure) |
| **Section 2.6 - MAJOR COMMENTS**: Biochemical stress markers were measured **only at Location 2** "due to logistic limitations" - this completely undermines comparison of stress physiology between locations, which is central to your hypothesis | Either: (1) Acknowledge this as a fatal limitation and remove claims about location-specific stress responses from results/discussion, or (2) Provide data from Location 1 |
| **Lines 142-143**: "A stick was attached to each plant" - vague; specify stake height and tying method | Add detail: "bamboo stakes (1.5 m height) placed 10 cm from each plant" |
| **Statistical analysis (Section 2.7)**: The LMM model selection (Table 2) shows Model 2 with AIC=291.88 and Model 1 with AIC=338.73 - difference of 46.85, which is appropriate selection, but why was Model 3 (AIC=373.04) even considered? | Justify the three candidate models conceptually, not just statistically |

**5. Results**

| **Comments** | **Suggestion** |
| --- | --- |
| **Table 3 - CRITICAL ERRORS**: |  |
| *Line 1 of table*: "SPAD value" for I0 shows 85.8 ± 17.66 - but this same value appears in Table 4 for Location 2 I0? Check for data copying errors | Verify all values; Tables 3 and 4 have identical numbers for I0 row in SPAD column - this cannot be correct for different locations |
| *LAI row for ID*: "129.23 ± 14.3" but text says "highest LAI was measured under ID and lowest LAI was observed at ID" - **contradictory/typo** | Correct: likely one of these is IC or IO |
| *Table 3 interaction section*: IC×MS shows SPAD 46.17 ± 4.19 (extremely low) while other IC combinations are higher - any explanation for this specific combination failing? | Discuss possible measurement error or pest damage at this specific plot |
| **Figure 3**: Multiple panels (H2O2, MDA, CAT, APX, Proline) - the figure quality is poor; letters for significance are barely visible | Reformat with larger font for significance indicators; ensure consistent scaling across panels |
| **Section 3.3.1 Soil moisture content - DATA ERROR**: Line 399 shows "I0 (22.27±3.63%)" at location 1 - but rainfed treatment should have *lowest* moisture, yet IC (27.62%) and ID (27.36%) are similar. ID should have higher moisture than IC if designed properly | Explain why drip irrigation did NOT maintain higher soil moisture than furrow irrigation at location 1 |
| **Section 3.4 Brinjal yield - CRITICAL**: Figure 7 shows ID×MP at location 1 yielding ~15 t ha⁻¹, but Table 5 (IWUE) shows ID×Mp = 16.56 kg ha⁻¹ mm⁻¹ - these numbers don't align with yield calculations | Recalculate IWUE values and verify against actual yield data |
| **Line 463**: "IC×MP produced highest yield (23.18±6.62) at location 1, statistically similar with ID×MS (12.93±4.53)" - a difference of 10 t ha⁻¹ cannot be "statistically similar" unless your test is underpowered | Report the actual p-value or confidence interval for this comparison |

**6. Discussion**

| **Comments** | **Suggestion** |
| --- | --- |
| **Lines 532-536**: Claim that "SPAD value was higher in non-irrigated plot compared to drip irrigation... due to smaller, thicker leaves" - but you have no leaf thickness or specific leaf area data to support this mechanism | Either add these measurements or rephrase as speculation |
| **Lines 550-554**: Citing studies showing 14-50% IWUE improvement with drip irrigation - but your location 1 showed drip underperforming conventional irrigation. This contradiction needs direct address | Add paragraph explaining why your results diverge from literature (soil texture, system design, irrigation scheduling differences) |
| **Lines 576-580**: "H2O2 values at location 1 suggests that drip irrigation was not comparable" - but you DID NOT MEASURE H2O2 at location 1 (only location 2). This is a **fatal overreach** | Remove any claims about location-specific stress marker differences; acknowledge the limitation explicitly |
| **Lines 595-597**: "Drip irrigation increased ~117% and ~32% fruit yield compared to no irrigation and conventional furrow at location 2" - but Table 4 shows ID (21.88) vs IC (14.68) = 49% increase, not 32%; recalculate | Provide transparent calculations: (21.88 - 10.1)/10.1 = 117% for IO; (21.88 - 14.68)/14.68 = 49% for IC, not 32% |
| **Lines 610-614**: The LMM interpretation is fine but overstates novelty - mixed models for agricultural field trials are standard | Tone down; simply state the findings without claiming methodological innovation |

**7. Tables and Figures**

| **Issue** | **Location** | **Suggestion** |
| --- | --- | --- |
| Table 1: Unit for EC is "S cm⁻¹" - should be "µS cm⁻¹" (microsiemens) | Line 104 | Correct units |
| Table 1: Potassium units "meq 100-g⁻¹ soil" - non-standard; use cmol kg⁻¹ | Line 105 | Convert to standard units |
| Table 3 & 4: Significance markers (*, **, NS) - inconsistent use; Table 3 shows "**" for irrigation effect on SPAD but text says "significant" - need legend explaining * = p<0.05, ** = p<0.01, *** = p<0.001 | All tables | Add explicit legend |
| Figure 1: Poor quality map; location boundaries unclear | Page 14 | Provide higher resolution image with clear labels |
| Figure 2: Weather data - temperature ribbon shows maximum and minimum, but rainfall data is missing from the figure despite being discussed | Page 16 | Add rainfall bars or clarify that rainfall was "insignificant" as stated |
| Figure 4,5,6,7: All use identical axis labels and formatting; letters for significance are too small and sometimes overlapping | Multiple | Standardize font sizes (minimum 10pt for significance letters) |
| Supplementary Figure 1: DHARMa diagnostic plots - the QQ plot shows some deviation at tails | Page 55 | Comment on whether this deviation is acceptable for inference |

**8. References**

| **Comments** | **Suggestion** |
| --- | --- |
| Reference 34 (BARC, 2018) - fertilizer recommendation guide - this is a national document but no specific page or table cited | Add specific section or table number |
| References 35, 36, 37, 38, 39 - methods papers from 1973-2021 - ensure extraction protocols are fully described in Methods, not just cited | Add critical details (centrifugation speed, incubation times, standard curve ranges) to Methods section |
| Reference 40 - "R Core Team, 2024" - incomplete; R version 4.4.1 has specific release date | Add: "R version 4.4.1 (2024-06-14)" |
| Missing citations for agronomic practices in brinjal (plant spacing, fertilizer rates specific to eggplant) | Add 2-3 key brinjal agronomy references from Bangladesh or similar contexts |

**9. Language and Presentation**

| **Problem** | **Example** | **Correction** |
| --- | --- | --- |
| Subject-verb agreement | Title "Responses...depends" | "Responses...depend" |
| Typo | Line 48 "Brinal" | "Brinjal" |
| Typo | Line 110 "location 2" (should be location 1) | Correct |
| Inconsistent terminology | "conventional alternate furrow irrigation" vs "conventional light furrow irrigation" | Use one term consistently |
| Redundancy | "approximately ~10.6 t ha⁻¹" (Line 22) | Remove "approximately" |
| Awkward phrasing | Line 456-457 "At location 2 the trend was found to be opposite, irrigation significantly boosted" | "At location 2, the opposite trend was observed; irrigation significantly increased yield" |
| Missing units | Line 151 "55 mL of water" - per what time? | "55 mL min⁻¹ plant⁻¹" |

**Specific Corrective Actions Required**

**Must-fix before resubmission:**

1. **Correct the contradictory yield data** in Tables 3-4 and Figure 7; ensure values match across all displays
2. **Either measure stress markers at Location 1** or **remove all location-comparison claims** about H2O2, MDA, CAT, APX, and proline
3. **Provide complete irrigation scheduling details** (frequency, duration, total water applied per treatment in mm)
4. **Fix the typo in Line 110** (location 2 → location 1)
5. **Replace "statistically similar" claims** with actual p-values and effect sizes (Cohen's d or standardized mean differences)
6. **Re-calculate percentage increases** in Discussion section (lines 595-597) to match actual data
7. **Add CV values to all tables** - Table 4 shows CV but Table 3 also has CV (bottom row) - ensure consistent reporting

**Strongly recommended:**

8. **Add a limitations section** acknowledging: (a) only two locations without true replication, (b) stress markers measured at only one site, (c) high variance in many measurements suggesting possible measurement error or insufficient sample size
9. **Report the statistical power** of your comparisons given the small sample size (n=3 replications per treatment combination)
10. **Provide raw data** as supplementary material to allow verification of the unusually high standard deviations

**Verdict**

**Major Revision Required**

The manuscript addresses an important question but suffers from:

- Data inconsistencies between text, tables, and figures
- Incomplete methodological reporting (irrigation amounts, stress marker protocols)
- A critical design limitation (stress markers at only one location)
- Overstated conclusions not supported by the data

The findings regarding plastic mulching are robust and valuable, but the irrigation × soil interaction claims require substantially stronger evidence. The authors should either complete the missing measurements or recalibrate their claims to match what the data actually support.

**Recommendation:** Return to authors for major revision (minimum 3 months) with mandatory re-analysis and either collection of missing data or transparent acknowledgment of limitations
