# Supplementary material for: Plastic mulching consistently improves brinjal performance, but drip irrigation benefits are soil dependent: A two-location field study
Source: PLoS One. 2026 Oct 27;21(10):e0360413. doi: 10.1371/journal.pone.0360413 (PMC13712550; doi:10.1371/journal.pone.0360413)
Supplement: Supplementary file 2 [file pone.0360413.s005.docx]

**Responses to PLOS ONE Technical Requirements**

**Manuscript: PONE-D-26-17291**

**Title: "Responses of brinjal to irrigation and mulching treatments depends on soil characteristics"**

**Requirement 1: PLOS ONE style formatting**
We have followed the PLOS ONE style templates and formatted our manuscript accordingly.

**Requirement 2: Author generated software or code**
No author-generated software or custom code underpins the findings of this study. The analyses were conducted using ‘R’ (version 4.4.1) with standard statistical procedures described in the Materials and Methods section.

**Requirement 3–4: Funding and funder role**
The Funding Statement has been amended to identify the only source of support received for the study and to confirm that there was no additional external funding. The role-of-funder statement has also been added to the revised cover letter.

**Requirement 5: Competing interests**
The competing-interests declaration has been amended to read: “The authors have declared that no competing interests exist.”

**Requirement 6: Data availability**
The replicate-level minimal dataset underlying all reported findings has been uploaded as S1 Data. The Data Availability Statement has been revised accordingly.

**Requirement 7: Funding in Acknowledgments**
Funding-related information has been removed from the Acknowledgments section.

**Requirement 8: Figure 1**
Figure 1 was redrawn by the authors using OpenStreetMap data only. No proprietary Google or other copyrighted map imagery is included. Appropriate attribution and Open Database License information have been added to the figure caption.

**Requirement 8: Citation**
The reviewer didn’t ask to cite specific reference.

**Professional Academic Peer Review**

**Manuscript: PONE-D-26-17291**

**Title: "Responses of brinjal to irrigation and mulching treatments depends on soil characteristics"**

**Overall Assessment**

This manuscript presents a field study examining the interactive effects of irrigation regimes and mulching practices on brinjal (eggplant) growth, yield, and physiological responses across two contrasting soil types in Bangladesh. The research addresses an important topic in water-scarce agriculture. However, the manuscript contains **significant methodological flaws, data inconsistencies, and presentation errors** that undermine its scientific rigor and clarity. **Major revisions are required before publication can be considered.**

**Major Critical Comments**

**1. Title**

| **Comments** | **Suggestion** |  |
| --- | --- | --- |
| Grammatical error: "depends" should be "depend" (subject-verb agreement with plural "responses") | Revise to: **"Responses of brinjal to irrigation and mulching treatments depend on soil characteristics"** | Thank you for pointing the mistake. We have used the suggested alternate title. |
| Title is descriptive but lacks specificity about the key finding | Consider: **"Plastic mulching consistently improves brinjal performance, but drip irrigation benefits are soil-dependent: A two-location field study"** | Thank you for suggesting this title which is much befitting to the content of the manuscript. We have accepted and used this title. |

**2. Abstract**

| **Comments** | **Suggestion** |  |
| --- | --- | --- |
| **Critical contradiction**: Line 15 reports drip irrigation yield at Location 1 as 8.9 ± 5.68 t ha⁻¹ (SD = 64% of mean!), yet Line 19 shows 23.18 ± 6.62 t ha⁻¹ under IC×MP. The enormous SD suggests extreme variability or measurement errors | Report coefficients of variation and explain why drip irrigation performed so poorly at Location 1; consider whether the drip system was appropriately designed for sandy soil | We thank the reviewer for noting the large standard deviation of fruit yield under drip irrigation at Location 1. The raw data were examined and the reported mean and standard deviation are correct. The relatively high standard deviation suggests that there is quite a bit of variation among the three replicate plots under this treatment. This variability was more pronounced in Location 1, which had coarser soil, lower organic matter and nutrient contents and lower water holding capacity than Location 2. This heterogeneity in the field may have resulted in patchy soil-water availability and thus variable responses of plants across replicate plots.  We have also reported that the experimental coefficient of variation for fruit yield for Location 1 was 20.58%. This value is the residual variation across the experiment and should not be interpreted as the treatment-specific coefficient of variation calculated directly from the mean and standard deviation of the drip-irrigation treatment. The treatment standard deviation may also have been strongly influenced by differences among individual plots, because only three replications were included. We have thus identified this variability as a limitation and have adjusted the interpretation of the Location 1 results. |
| Line 16 states drip irrigation at Location 1 was "statistically similar to IC and IO" - with such high variance, this conclusion is statistically weak; report actual p-values or confidence intervals | Replace vague "statistically similar" with precise statistical metrics | We have added the p value (p = 0.88). |
| The abstract claims "plastic mulching consistently enhanced brinjal performance" but Line 23 shows Model estimated +10.6 t ha⁻¹ - this needs confidence intervals | Report 95% CI around this estimate | We have added the CI. In addition, we have added the CI for all other estimates as a new column to Table 7. |
| Missing key information: duration of study, actual irrigation amounts applied, and explanation of why Location 1 had *lower* yield under drip than rainfed? (8.9 vs not specified for IO) | Add comparative rainfed yield values |  |

**3. Introduction**

| **Comments** | **Suggestion** |  |
| --- | --- | --- |
| **Line 48**: "Brinal" typo - should be "Brinjal" | Correct throughout manuscript | We have corrected this typo and checked the manuscript thoroughly for other typos. |
| Lines 29-31: General statements about global water scarcity lack specific citations to brinjal production contexts | Add region-specific statistics for brinjal water requirements in South Asia | We have added some region-specific water requirements for brinjal. |
| Lines 53-56: Claim that drip irrigation "increases water productivity and yield compared to conventional furrow" is overly simplistic given your own findings (Location 1 showed no benefit) | Acknowledge early that soil type moderates this response | The previous paragraph, described this statement with citation. |
| Lines 75-78: The research gap is framed as "performance in contrasting soil types not well documented" - but your own methods lack replication across soil types (only 2 locations, not a gradient) | Reframe as "preliminary comparison between two distinct soil textures" | We have rephrased it. |
| Missing: Justification for the specific irrigation levels (why 20-day intervals for conventional irrigation?) | Add rationale based on local practice or preliminary studies | Conventional furrow irrigation was applied at 20-day intervals to represent the less-frequent end of the traditional farmer-managed surface-irrigation practice used for brinjal production in Bangladesh. This treatment was included as a practical conventional-irrigation benchmark for comparison with drip irrigation rather than as an optimized irrigation schedule.  We have added this justification in the revised manuscript. |

**4. Materials and Methods**

| **Comments** | **Suggestion** |  |
| --- | --- | --- |
| **Critical flaw - Section 2.1, Table 1**: Location 1 is described as "sandy loam" with particle density 2.79 g cc⁻¹, Location 2 as "silt loam" with 2.63 g cc⁻¹ - but **no replication of locations** (each soil type has only ONE field). This confounds soil type with other site-specific factors (history, management, microclimate) | Acknowledge this as a severe limitation in the Discussion; state clearly that "location" is a proxy for soil type, not a true treatment | We thank the reviewer for highlighting this important limitation. We agree that each soil texture was represented by only one field location and, soil type was not independently replicated. Therefore, the effects of soil texture cannot be separated from other location-specific factors, such as previous land use, management history, soil fertility, microclimate, and pest or disease pressure.  In the discussion of the revised manuscript, we have clearly stated that location was used only as a proxy for the contrasting soil conditions and it should not be considered a true replicated soil-type treatment. |
| **Line 110**: "Location 2 has a silt loam soil which means better water holding capacity compared to the sandy loam of location 2" - **This is a typo** (should be "location 1") | Correct: "compared to the sandy loam of location 1" | We have corrected this typo. Thank you. |
| **Section 2.3**: Irrigation amounts are inadequately described. "55 mL of water was applied per minute per plant" -这是什么意思? This does not translate to mm without knowing plant spacing and emitter configuration | Provide: emitter flow rate (L h⁻¹), number of emitters per plant, irrigation duration and frequency, total applied water (mm) for each treatment | We have added much elaborated description of the drip irrigation system. |
| **Line 140**: "Irrigation was given regularly automatically with drip irrigation system" - vague; specify controller settings, pressure, and verification method | Add technical specifications of the drip system (manufacturer, emitter spacing, operating pressure) | We have added much elaborated description of the drip irrigation system. |
| **Section 2.6 - MAJOR COMMENTS**: Biochemical stress markers were measured **only at Location 2** "due to logistic limitations" - this completely undermines comparison of stress physiology between locations, which is central to your hypothesis | Either: (1) Acknowledge this as a fatal limitation and remove claims about location-specific stress responses from results/discussion, or (2) Provide data from Location 1 | We thank the reviewer for identifying this important limitation. We agree that measuring the biochemical stress markers only at Location 2 prevents a valid comparison of stress physiology between the two locations. Since corresponding biochemical data were not collected at Location 1, the effects of location, soil condition, and their interactions with irrigation and mulching on plant stress physiology cannot be evaluated.  In fact, anywhere in the manuscript, we did not make any inference on location-specific differences in biochemical stress responses. Rather, the biochemical data are presented and interpreted exclusively as Location 2–specific observations. The biochemical measurements are retained as supplementary physiological evidence of treatment responses at Location 2, but they were not used to explain differences between the two field locations or to support conclusions regarding the influence of contrasting soil conditions on plant stress physiology.  This constraint has been explicitly acknowledged as a major limitation in the revised Discussion. We further state that a robust assessment of location- or soil-dependent stress physiology would require the same biochemical measurements to be conducted at all locations under a replicated multi-site design. |
| **Lines 142-143**: "A stick was attached to each plant" - vague; specify stake height and tying method | Add detail: "bamboo stakes (1.5 m height) placed 10 cm from each plant" | We have revise here as suggested. |
| **Statistical analysis (Section 2.7)**: The LMM model selection (Table 2) shows Model 2 with AIC=291.88 and Model 1 with AIC=338.73 - difference of 46.85, which is appropriate selection, but why was Model 3 (AIC=373.04) even considered? | Justify the three candidate models conceptually, not just statistically | Model 1 was specified to estimate the overall effects of irrigation, mulch, and their interaction across the two study locations, while accounting for the hierarchical structure of replications nested within location. In this formulation, location was treated as a grouping factor, and the treatment effects were assumed to be common across locations.  Model 2 was specified to determine whether irrigation and mulch responses were location-specific. Location was therefore included as a fixed factor together with all treatment-by-location interactions. This model allowed the treatment means and treatment effects to differ explicitly between the two experimental sites.  Model 3 was considered as an exploratory model to evaluate whether the effects of irrigation, mulch, and their interaction varied among locations by allowing treatment-specific random slopes for location. Conceptually, this formulation represented heterogeneity in treatment responses among sites rather than estimating separate fixed treatment effects for each location.  However, including this discussion would make the manuscript too long. Therefore, we have not added the discussion in the manuscript. |

**5. Results**

| **Comments** | **Suggestion** |  |
| --- | --- | --- |
| **Table 3 - CRITICAL ERRORS**: |  |  |
| *Line 1 of table*: "SPAD value" for I0 shows 85.8 ± 17.66 - but this same value appears in Table 4 for Location 2 I0? Check for data copying errors | Verify all values; Tables 3 and 4 have identical numbers for I0 row in SPAD column - this cannot be correct for different locations | We apologize for the mistake. Yes, it was a typing mistake. We have corrected it. |
| *LAI row for ID*: "129.23 ± 14.3" but text says "highest LAI was measured under ID and lowest LAI was observed at ID" - **contradictory/typo** | Correct: likely one of these is IC or IO | Thank you for pointing it out. We have checked the entire manuscript again for this type of mistakes and corrected where they were found. |
| *Table 3 interaction section*: IC×MS shows SPAD 46.17 ± 4.19 (extremely low) while other IC combinations are higher - any explanation for this specific combination failing? | Discuss possible measurement error or pest damage at this specific plot | We have added the discussion as suggested. |
| **Figure 3**: Multiple panels (H2O2, MDA, CAT, APX, Proline) - the figure quality is poor; letters for significance are barely visible | Reformat with larger font for significance indicators; ensure consistent scaling across panels | We have redrawn the figure and replaced the old figure with high quality one. |
| **Section 3.3.1 Soil moisture content - DATA ERROR**: Line 399 shows "I0 (22.27±3.63%)" at location 1 - but rainfed treatment should have *lowest* moisture, yet IC (27.62%) and ID (27.36%) are similar. ID should have higher moisture than IC if designed properly | Explain why drip irrigation did NOT maintain higher soil moisture than furrow irrigation at location 1 | We rechecked the soil-moisture dataset and confirmed that the reported values were correctly entered. We agree that drip irrigation is often expected to provide better soil water availability in the active root zone. However, drip irrigation does not always result in greater measured bulk soil moisture than furrow irrigation, especially in coarse-textured soils with low organic matter and low water-holding capacity as seen at Location 1. Under such soil conditions, water applied by drip emitters may drain rapidly or remain concentrated in a limited wetted zone, while furrow irrigation may temporarily wet a larger volume of soil. Furthermore, the measured soil moisture depends on the timing of soil sampling after irrigation and the position and depth of soil sampling with respect to the drip emitter or furrow. |
| **Section 3.4 Brinjal yield - CRITICAL**: Figure 7 shows ID×MP at location 1 yielding ~15 t ha⁻¹, but Table 5 (IWUE) shows ID×Mp = 16.56 kg ha⁻¹ mm⁻¹ - these numbers don't align with yield calculations | Recalculate IWUE values and verify against actual yield data | We have rechecked the IWUE calculations against the fruit-yield data. Fruit yield and irrigation water use efficiency are not expected to have the same numerical values because they represent different parameters. Fruit yield is expressed as t ha⁻¹, whereas IWUE was calculated as fruit yield per unit of irrigation water applied, expressed as kg ha⁻¹ mm⁻¹. |
| **Line 463**: "IC×MP produced highest yield (23.18±6.62) at location 1, statistically similar with ID×MS (12.93±4.53)" - a difference of 10 t ha⁻¹ cannot be "statistically similar" unless your test is underpowered | Report the actual p-value or confidence interval for this comparison | We thank the reviewer for this important comment. We double checked pairwise comparison between IC x MP and ID x MS at Location 1. The pairwise comparison was not statistically significant at the 5% level (p = 0.0789) despite a large numerical difference between the two treatment means. Thus, the post hoc comparison result was the basis for the statement that these treatments were statistically similar.  But we agree that the large numerical difference warrants more explicit reporting and cautious interpretation. In the revised manuscript we have added the actual pairwise p-value and stressed that the comparison showed a nonsignificant trend instead of a definite equality between treatments. We also observed a relatively high experimental variability (CV = 20.86 %) which may have been responsible for the lack of statistical significance despite the large numerical difference. |

**6. Discussion**

| **Comments** | **Suggestion** |  |
| --- | --- | --- |
| **Lines 532-536**: Claim that "SPAD value was higher in non-irrigated plot compared to drip irrigation... due to smaller, thicker leaves" - but you have no leaf thickness or specific leaf area data to support this mechanism | Either add these measurements or rephrase as speculation | We have revised the discussion to say that the higher SPAD value in non-irrigated conditions may indicate changes in the leaf chlorophyll concentration or other physiological responses related to the treatment. However, the underlying mechanism could not be determined by the present data because the leaf thickness, specific leaf area and chlorophyll concentration were not measured directly. This interpretation is now stated explicitly to be speculative, and the lack of such supporting measurements has been acknowledged. |
| **Lines 550-554**: Citing studies showing 14-50% IWUE improvement with drip irrigation - but your location 1 showed drip underperforming conventional irrigation. This contradiction needs direct address | Add paragraph explaining why your results diverge from literature (soil texture, system design, irrigation scheduling differences) | We thank the reviewer for the pointing out this inconsistency. We agree that previous studies reported improved IWUE under drip irrigation however, our result at Location 1 showed that drip irrigation did not perform better than conventional furrow irrigation. We have altered the Discussion to explicitly address this deviation. |
| **Lines 576-580**: "H2O2 values at location 1 suggests that drip irrigation was not comparable" - but you DID NOT MEASURE H2O2 at location 1 (only location 2). This is a **fatal overreach** | Remove any claims about location-specific stress marker differences; acknowledge the limitation explicitly | We have responded to this issue in the method section. We have added pertaining discussion and limitation. |
| **Lines 595-597**: "Drip irrigation increased ~117% and ~32% fruit yield compared to no irrigation and conventional furrow at location 2" - but Table 4 shows ID (21.88) vs IC (14.68) = 49% increase, not 32%; recalculate | Provide transparent calculations: (21.88 - 10.1)/10.1 = 117% for IO; (21.88 - 14.68)/14.68 = 49% for IC, not 32% |  |
| **Lines 610-614**: The LMM interpretation is fine but overstates novelty - mixed models for agricultural field trials are standard | Tone down; simply state the findings without claiming methodological innovation | We have revised the text to tone down the interpretation and now report the LMM results simply as statistical evidence supporting the observed treatment effects. |

**7. Tables and Figures**

| **Issue** | **Location** | **Suggestion** |  |
| --- | --- | --- | --- |
| Table 1: Unit for EC is "S cm⁻¹" - should be "µS cm⁻¹" (microsiemens) | Line 104 | Correct units | We have corrected the unit to µS cm⁻¹ |
| Table 1: Potassium units "meq 100-g⁻¹ soil" - non-standard; use cmol kg⁻¹ | Line 105 | Convert to standard units | We have corrected the unit in table and in the text |
| Table 3 & 4: Significance markers (*, **, NS) - inconsistent use; Table 3 shows "**" for irrigation effect on SPAD but text says "significant" - need legend explaining * = p<0.05, ** = p<0.01, *** = p<0.001 | All tables | Add explicit legend | We have added legends to the respective tables. |
| Figure 1: Poor quality map; location boundaries unclear | Page 14 | Provide higher resolution image with clear labels | In our research, we did not conduct any spatia analysis with the location. We just wanted to point out the location in respect to Bangladesh. So, we used only point marker and did not show any boundary of the study sites. |
| Figure 2: Weather data - temperature ribbon shows maximum and minimum, but rainfall data is missing from the figure despite being discussed | Page 16 | Add rainfall bars or clarify that rainfall was "insignificant" as stated | Rainfall data is already there! The blue line represents rainfall, and the line has been annotated. |
| Figure 4,5,6,7: All use identical axis labels and formatting; letters for significance are too small and sometimes overlapping | Multiple | Standardize font sizes (minimum 10pt for significance letters) | We have redrawn all figures. We hope the label font size is uniform across the figures and there are no overlapping of the label markers. |
| Supplementary Figure 1: DHARMa diagnostic plots - the QQ plot shows some deviation at tails | Page 55 | Comment on whether this deviation is acceptable for inference | We appreciate you pointing out about a little tail deviation of residuals in the DHARMa diagnostic QQ plot. However, the ks test, dispersion test, and outlier test suggested that the deviation is non-significant. |

**8. References**

| **Comments** | **Suggestion** |  |
| --- | --- | --- |
| Reference 34 (BARC, 2018) - fertilizer recommendation guide - this is a national document but no specific page or table cited | Add specific section or table number | We have added the brinjal specific section and its page number in the reference. |
| References 35, 36, 37, 38, 39 - methods papers from 1973-2021 - ensure extraction protocols are fully described in Methods, not just cited | Add critical details (centrifugation speed, incubation times, standard curve ranges) to Methods section | We thank the reviewer for this helpful suggestion. We agree that additional methodological details are important for reproducibility. However, because these biochemical assays were performed following established published protocols, we have kept the main text concise to avoid excessive expansion of the Materials and Methods section. We have added the key assay conditions, including extraction medium, incubation, centrifugation speed etc. where applicable. The full procedural details are available in the cited original protocols. We believe this revision provides sufficient information for reproducibility while maintaining the readability and length of the manuscript. |
| Reference 40 - "R Core Team, 2024" - incomplete; R version 4.4.1 has specific release date | Add: "R version 4.4.1 (2024-06-14)" | We have added the version and date of R as suggested in the reference. |
| Missing citations for agronomic practices in brinjal (plant spacing, fertilizer rates specific to eggplant) | Add 2-3 key brinjal agronomy references from Bangladesh or similar contexts | The fertilizer rate was selected following the region-specific recommendation provided in the national Fertilizer Recommendation Guide of Bangladesh, which has now been cited appropriately (BARC, 2018).  Regarding plant spacing, the spacing used in this experiment followed the standard local cultivation practice commonly adopted by brinjal farmers in the study area. Because this spacing was based on prevailing farmer practice rather than a specific experimental protocol, no separate citation was originally provided. |

**9. Language and Presentation**

| **Problem** | **Example** | **Correction** |  |
| --- | --- | --- | --- |
| Subject-verb agreement | Title "Responses...depends" | "Responses...depend" | Corrected |
| Typo | Line 48 "Brinal" | "Brinjal" | Corrected |
| Typo | Line 110 "location 2" (should be location 1) | Correct | Corrected |
| Inconsistent terminology | "conventional alternate furrow irrigation" vs "conventional light furrow irrigation" | Use one term consistently | Corrected |
| Redundancy | "approximately ~10.6 t ha⁻¹" (Line 22) | Remove "approximately" | Corrected |
| Awkward phrasing | Line 456-457 "At location 2 the trend was found to be opposite, irrigation significantly boosted" | "At location 2, the opposite trend was observed; irrigation significantly increased yield" | Corrected |
| Missing units | Line 151 "55 mL of water" - per what time? | "55 mL min⁻¹ plant⁻¹" |  |

**Specific Corrective Actions Required**

**Must-fix before resubmission:**

1. **Correct the contradictory yield data** in Tables 3-4 and Figure 7; ensure values match across all displays
2. **Either measure stress markers at Location 1** or **remove all location-comparison claims** about H2O2, MDA, CAT, APX, and proline
3. **Provide complete irrigation scheduling details** (frequency, duration, total water applied per treatment in mm)
4. **Fix the typo in Line 110** (location 2 → location 1)
5. **Replace "statistically similar" claims** with actual p-values and effect sizes (Cohen's d or standardized mean differences)
6. **Re-calculate percentage increases** in Discussion section (lines 595-597) to match actual data
7. **Add CV values to all tables** - Table 4 shows CV but Table 3 also has CV (bottom row) - ensure consistent reporting

**Strongly recommended:**

8. **Add a limitations section** acknowledging: (a) only two locations without true replication, (b) stress markers measured at only one site, (c) high variance in many measurements suggesting possible measurement error or insufficient sample size
9. **Report the statistical power** of your comparisons given the small sample size (n=3 replications per treatment combination)
10. **Provide raw data** as supplementary material to allow verification of the unusually high standard deviations

**Verdict**

**Major Revision Required**

The manuscript addresses an important question but suffers from:

- Data inconsistencies between text, tables, and figures
- Incomplete methodological reporting (irrigation amounts, stress marker protocols)
- A critical design limitation (stress markers at only one location)
- Overstated conclusions not supported by the data

The findings regarding plastic mulching are robust and valuable, but the irrigation × soil interaction claims require substantially stronger evidence. The authors should either complete the missing measurements or recalibrate their claims to match what the data actually support.

**Recommendation:** Return to authors for major revision (minimum 3 months) with mandatory re-analysis and either collection of missing data or transparent acknowledgment of limitations
