# Supplementary material for: Plastic mulching consistently improves brinjal performance, but drip irrigation benefits are soil dependent: A two-location field study
Source: PLoS One. 2026 Oct 27;21(10):e0360413. doi: 10.1371/journal.pone.0360413 (PMC13712550; doi:10.1371/journal.pone.0360413)
Supplement: Supplementary file 3 [file pone.0360413.s006.docx]

**Manuscript: PONE-D-26-17291R1**

**Title: "Plastic mulching consistently improves brinjal performance, but drip irrigation benefits are soil dependent: A two-location field study"**

Dear Academic Editor and Reviewers,

We sincerely thank the Academic Editor and the reviewers for their careful evaluation of our revised manuscript. We appreciate the positive assessment of the revisions made during the previous round and the opportunity to address the remaining points.

All applicable comments have been carefully addressed in the revised manuscript. Our point-by-point responses are provided below.

**Response to the Academic Editor**

**Comment:**

*Please incorporate all the comments recommended by reviewers and check complete MS for minor errors.*

**Response:**

Thank you for the guidance. The complete manuscript has been carefully checked again for typographical, grammatical, and presentation-related errors. All four remaining minor corrections identified by Reviewer 1 have been incorporated into the revised manuscript. We have also carefully examined the comments attributed to Reviewer 2. As explained below, those comments appear to concern a different manuscript and therefore could not appropriately be incorporated into the present study.

**Reviewer 1**

We sincerely thank Reviewer 1 for reassessing our revised manuscript and for confirming that the previous comments have been adequately addressed. We also appreciate the identification of the four remaining minor issues. Reviewer 1 assessed the work as technically sound and statistically appropriate and recommended publication following these corrections.

**Comment 1:** *Abstract typo: “IC and IC” → “IC and I₀” (Line 17, Page 10).*

**Response:**

Thank you for identifying this typographical error. In fact, the drip irrigation outperformed the control (I_0_) by 117% and the conventional furrow irrigation (I_C_) to 32%. Therefore, in accoradnace with the result, the text has been corrected from **“IC and IC”** to **“I_0_ and I_C_”** in the Abstract.

**Comment 2:** *LAI contradiction: Lowest LAI at Location 1 should be under I₀, not I_D (Lines 313–317, Page 26).*

**Response:**

Thank you for identifying this inconsistency. The statement has been corrected to indicate that the **lowest LAI at Location 1 was recorded under I₀**, in agreement with the corresponding results.

**Comment 3:** *Grammar: “At both the locations” → “At both locations” (Line 578, Page 95).*

**Response:**

Thank you. The phrase has been corrected from **“At both the locations”** to **“At both locations.”**

**Comment 4:** *Grammar: “H₂O₂ values...suggests” → “suggest” (Line 638, Page 98).*

**Response:**

Thank you for identifying the grammatical error. The verb has been corrected from **“suggests”** to **“suggest”** to agree with the plural subject **“H₂O₂ values.”**

**Reviewer 2**

We thank Reviewer 2 for evaluating the manuscript. We note that Reviewer 2 answered **“Yes”** to the questions concerning technical soundness, appropriateness and rigor of the statistical analysis, availability of underlying data, and presentation in standard English.

**Comment:** The reviewer comments on screening resilient plants and waste-derived substrates for vertical greenery systems, *Buxus sinica*, a 3:1:1 garden-waste:straw:biogas-residue substrate, drought and waterlogging treatments, alternating stresses, and a fuzzy membership model.

**Response**

Thank you for the assessment. We have carefully examined this comment. However, we respectfully believe that the comment may have been **inadvertently associated with the present manuscript**, as the topics and experimental details described in the comment do not correspond to our study.

The present manuscript, entitled *“Plastic Mulching Consistently Improves Brinjal Performance, but Drip Irrigation Benefits Are Soil Dependent: A Two-Location Field Study,”* investigates the effects of **plastic mulching and drip irrigation on brinjal under field conditions at two locations**. It does not involve vertical greenery systems, *Buxus sinica*, waste-derived growth substrates, drought and waterlogging screening, alternating stress treatments, or fuzzy membership modelling. In contrast, all of these elements are specifically referred to in the comment attributed to Reviewer 2.

Consequently, we are unable to make scientifically appropriate revisions to the present manuscript on the basis of these comments because the issues raised do not relate to its objectives, experimental design, treatments, analyses, or findings.

We respectfully request the Academic Editor to consider this apparent discrepancy. **If Reviewer 2 has any additional comments or recommendations specifically related to the present manuscript, we would be very pleased to receive them and will address them carefully and comprehensively.**

We thank the reviewers and the Academic Editor for their consideration.
