# Supplementary material for: Plastic mulching consistently improves brinjal performance, but drip irrigation benefits are soil dependent: A two-location field study
Source: PLoS One. 2026 Oct 27;21(10):e0360413. doi: 10.1371/journal.pone.0360413 (PMC13712550; doi:10.1371/journal.pone.0360413)
Supplement: Peer review history

# Peer review: Decision Letter 0

**DOI:** 10.1371/journal.pone.0360413.r001

**Authors:** Umesh Sharma

1 Jun 2026

Dear Dr. Rashid,

Thank you for submitting your manuscript to PLOS ONE. After careful consideration, we feel that it has merit but does not fully meet PLOS ONE’s publication criteria as it currently stands. Therefore, we invite you to submit a revised version of the manuscript that addresses the points raised during the review process.

Please submit your revised manuscript by Jul 16 2026 11:59PM. If you will need more time than this to complete your revisions, please reply to this message or contact the journal office at plosone@plos.org. When you're ready to submit your revision, log on to https://www.editorialmanager.com/pone/ and select the 'Submissions Needing Revision' folder to locate your manuscript file.

- A letter that responds to each point raised by the academic editor and reviewer(s). You should upload this letter as a separate file labeled 'Response to Reviewers'.
- A marked-up copy of your manuscript that highlights changes made to the original version. You should upload this as a separate file labeled 'Revised Manuscript with Track Changes'.
- An unmarked version of your revised paper without tracked changes. You should upload this as a separate file labeled 'Manuscript'.

If you would like to make changes to your financial disclosure, please include your updated statement in your cover letter. Guidelines for resubmitting your figure files are available below the reviewer comments at the end of this letter.

If applicable, we recommend that you deposit your laboratory protocols in protocols.io to enhance the reproducibility of your results. Protocols.io assigns your protocol its own identifier (DOI) so that it can be cited independently in the future. For instructions see: https://journals.plos.org/plosone/s/submission-guidelines#loc-laboratory-protocols. Additionally, PLOS ONE offers an option for publishing peer-reviewed Lab Protocol articles, which describe protocols hosted on protocols.io. Read more information on sharing protocols at https://plos.org/protocols?utm_medium=editorial-email&utm_source=authorletters&utm_campaign=protocols.

As the corresponding author, your ORCID iD is verified in the submission system and will appear in the published article. PLOS supports the use of ORCID, and we encourage all coauthors to register for an ORCID iD and use it as well. Please encourage your coauthors to verify their ORCID iD within the submission system before final acceptance, as unverified ORCID iDs will not appear in the published article. *Only*  the individual author can complete the verification step; PLOS staff *cannot*  verify ORCID iDs on behalf of authors.

We look forward to receiving your revised manuscript.

Kind regards,

Umesh Sharma

Academic Editor

PLOS One

Journal Requirements:

When submitting your revision, we need you to address these additional requirements.

1. Please ensure that your manuscript meets PLOS ONE's style requirements, including those for file naming. The PLOS ONE style templates can be found at

https://journals.plos.org/plosone/s/file?id=wjVg/PLOSOne_formatting_sample_main_body.pdf and

https://journals.plos.org/plosone/s/file?id=ba62/PLOSOne_formatting_sample_title_authors_affiliations.pdf.

2. Please note that PLOS One has specific guidelines on code sharing for submissions in which author-generated code underpins the findings in the manuscript. In these cases, we expect all author-generated code to be made available without restrictions upon publication of the work. Please review our guidelines at https://journals.plos.org/plosone/s/materials-and-software-sharing#loc-sharing-code and ensure that your code is shared in a way that follows best practice and facilitates reproducibility and reuse.

3. Thank you for stating in your Funding Statement:

“The research was financial supported by the Australian Centre for International Agricultural Research (ACIAR) through the ‘RUPANTAR’ project (WAC/2020/148 Transforming smallholder food systems in the Eastern Gangetic Plains). “

Please provide an amended statement that declares *all* the funding or sources of support (whether external or internal to your organization) received during this study, as detailed online in our guide for authors at http://journals.plos.org/plosone/s/submit-now.  Please also include the statement “There was no additional external funding received for this study.” in your updated Funding Statement.

Please include your amended Funding Statement within your cover letter. We will change the online submission form on your behalf.

4.Thank you for stating the following financial disclosure:

“The research was financial supported by the Australian Centre for International Agricultural Research (ACIAR) through the ‘RUPANTAR’ project (WAC/2020/148 Transforming smallholder food systems in the Eastern Gangetic Plains). “

Please state what role the funders took in the study.  If the funders had no role, please state: "The funders had no role in study design, data collection and analysis, decision to publish, or preparation of the manuscript."

If this statement is not correct you must amend it as needed.

Please include this amended Role of Funder statement in your cover letter; we will change the online submission form on your behalf.

5. Thank you for stating the following in your Competing Interests section:

“None”

Please complete your Competing Interests on the online submission form to state any Competing Interests. If you have no competing interests, please state "The authors have declared that no competing interests exist.", as detailed online in our guide for authors at http://journals.plos.org/plosone/s/submit-now

This information should be included in your cover letter; we will change the online submission form on your behalf.

6. We note that your Data Availability Statement is currently as follows: [All relevant data are within the manuscript and its Supporting Information files. ]

Please confirm at this time whether or not your submission contains all raw data required to replicate the results of your study. Authors must share the “minimal data set” for their submission. PLOS defines the minimal data set to consist of the data required to replicate all study findings reported in the article, as well as related metadata and methods (https://journals.plos.org/plosone/s/data-availability#loc-minimal-data-set-definition).

For example, authors should submit the following data:

- The values behind the means, standard deviations and other measures reported;

- The values used to build graphs;

- The points extracted from images for analysis.

Authors do not need to submit their entire data set if only a portion of the data was used in the reported study.

If your submission does not contain these data, please either upload them as Supporting Information files or deposit them to a stable, public repository and provide us with the relevant URLs, DOIs, or accession numbers. For a list of recommended repositories, please see https://journals.plos.org/plosone/s/recommended-repositories.

If there are ethical or legal restrictions on sharing a de-identified data set, please explain them in detail (e.g., data contain potentially sensitive information, data are owned by a third-party organization, etc.) and who has imposed them (e.g., an ethics committee). Please also provide contact information for a data access committee, ethics committee, or other institutional body to which data requests may be sent. If data are owned by a third party, please indicate how others may request data access.

7. Thank you for stating the following in the Acknowledgments Section of your manuscript:

“The research was financial supported by the Australian Centre for International Agricultural Research (ACIAR) through the ‘RUPANTAR’ project (WAC/2020/148 Transforming smallholder food systems in the Eastern Gangetic Plains).”

We note that you have provided additional information within the Acknowledgements Section that is not currently declared in your Funding Statement. Please note that funding information should not appear in the Acknowledgments section or other areas of your manuscript. We will only publish funding information present in the Funding Statement section of the online submission form.

Please remove any funding-related text from the manuscript and let us know how you would like to update your Funding Statement. Currently, your Funding Statement reads as follows:

“The research was financial supported by the Australian Centre for International Agricultural Research (ACIAR) through the ‘RUPANTAR’ project (WAC/2020/148 Transforming smallholder food systems in the Eastern Gangetic Plains).”

Please include your amended statements within your cover letter; we will change the online submission form on your behalf.

8. We note that Figure 1 in your submission contain [map/satellite] images which may be copyrighted. All PLOS content is published under the Creative Commons Attribution License (CC BY 4.0), which means that the manuscript, images, and Supporting Information files will be freely available online, and any third party is permitted to access, download, copy, distribute, and use these materials in any way, even commercially, with proper attribution. For these reasons, we cannot publish previously copyrighted maps or satellite images created using proprietary data, such as Google software (Google Maps, Street View, and Earth). For more information, see our copyright guidelines: http://journals.plos.org/plosone/s/licenses-and-copyright.

We require you to either (1) present written permission from the copyright holder to publish these figures specifically under the CC BY 4.0 license, or (2) remove the figures from your submission:

a. You may seek permission from the original copyright holder of Figure 1 to publish the content specifically under the CC BY 4.0 license.

We recommend that you contact the original copyright holder with the Content Permission Form (http://journals.plos.org/plosone/s/file?id=7c09/content-permission-form.pdf) and the following text:

“I request permission for the open-access journal PLOS ONE to publish XXX under the Creative Commons Attribution License (CCAL) CC BY 4.0 (http://creativecommons.org/licenses/by/4.0/). Please be aware that this license allows unrestricted use and distribution, even commercially, by third parties. Please reply and provide explicit written permission to publish XXX under a CC BY license and complete the attached form.”

Please upload the completed Content Permission Form or other proof of granted permissions as an "Other" file with your submission.

In the figure caption of the copyrighted figure, please include the following text: “Reprinted from [ref] under a CC BY license, with permission from [name of publisher], original copyright [original copyright year].”

b. If you are unable to obtain permission from the original copyright holder to publish these figures under the CC BY 4.0 license or if the copyright holder’s requirements are incompatible with the CC BY 4.0 license, please either i) remove the figure or ii) supply a replacement figure that complies with the CC BY 4.0 license. Please check copyright information on all replacement figures and update the figure caption with source information. If applicable, please specify in the figure caption text when a figure is similar but not identical to the original image and is therefore for illustrative purposes only.

The following resources for replacing copyrighted map figures may be helpful:

USGS National Map Viewer (public domain): http://viewer.nationalmap.gov/viewer/

The Gateway to Astronaut Photography of Earth (public domain): http://eol.jsc.nasa.gov/sseop/clickmap/

Maps at the CIA (public domain): https://www.cia.gov/library/publications/the-world-factbook/index.html and https://www.cia.gov/library/publications/cia-maps-publications/index.html

NASA Earth Observatory (public domain): http://earthobservatory.nasa.gov/

Landsat: http://landsat.visibleearth.nasa.gov/

USGS EROS (Earth Resources Observatory and Science (EROS) Center) (public domain): http://eros.usgs.gov/#

Natural Earth (public domain): http://www.naturalearthdata.com/

9. If the reviewer comments include a recommendation to cite specific previously published works, please review and evaluate these publications to determine whether they are relevant and should be cited. There is no requirement to cite these works unless the editor has indicated otherwise.

Additional Editor Comments (if provided):

Dear Author,

At present, we have received comments from one reviewer. However, despite several invitations, we have not yet received acceptance from additional experts to review the revised manuscript. Based on the available review report, we are proceeding with the evaluation process and will continue our efforts to secure further referee assessments for the manuscript.

Reviewers' comments:

Reviewer's Responses to Questions

**Comments to the Author**

1. Is the manuscript technically sound, and do the data support the conclusions?

Reviewer #1: Yes

**********

2. Has the statistical analysis been performed appropriately and rigorously?

Reviewer #1: Yes

**********

3. Have the authors made all data underlying the findings in their manuscript fully available?

The PLOS Data policy

Reviewer #1: Yes

**********

4. Is the manuscript presented in an intelligible fashion and written in standard English?

Reviewer #1: Yes

**********

Reviewer #1: Peer Review Comments :

Title

Grammatical error: "depends" should be "depend" (subject-verb agreement with plural "responses")

Title is descriptive but lacks specificity about the key finding

Abstract

Critical contradiction: Line 15 reports drip irrigation yield at Location 1 as 8.9 ± 5.68 t ha⁻¹ (SD = 64% of mean!), yet Line 19 shows 23.18 ± 6.62 t ha⁻¹ under IC×MP

Line 16 states drip irrigation at Location 1 was "statistically similar to IC and IO" - with such high variance, this conclusion is statistically weak

The abstract claims "plastic mulching consistently enhanced brinjal performance" but Line 23 shows Model estimated +10.6 t ha⁻¹ - this needs confidence intervals

Missing key information: duration of study, actual irrigation amounts applied, and explanation of why Location 1 had lower yield under drip than rainfed

Introduction

Line 48: "Brinal" typo - should be "Brinjal"

Lines 29-31: General statements about global water scarcity lack specific citations to brinjal production contexts

Lines 53-56: Claim that drip irrigation "increases water productivity and yield compared to conventional furrow" is overly simplistic given your own findings (Location 1 showed no benefit)

Lines 75-78: The research gap is framed as "performance in contrasting soil types not well documented" - but your own methods lack replication across soil types (only 2 locations, not a gradient)

Missing justification for the specific irrigation levels (why 20-day intervals for conventional irrigation?)

Materials and Methods

Critical flaw - Section 2.1, Table 1: Location 1 is "sandy loam" with particle density 2.79 g cc⁻¹, Location 2 as "silt loam" with 2.63 g cc⁻¹ - but no replication of locations (each soil type has only ONE field). This confounds soil type with other site-specific factors.

Line 110: "Location 2 has a silt loam soil which means better water holding capacity compared to the sandy loam of location 2" - This is a typo (should be "location 1")

Section 2.3: Irrigation amounts are inadequately described. "55 mL of water was applied per minute per plant" does not translate to mm without knowing plant spacing and emitter configuration

Line 140: "Irrigation was given regularly automatically with drip irrigation system" - vague; no controller settings, pressure, or verification method provided

Section 2.6 - MAJOR WEAKNESS: Biochemical stress markers were measured only at Location 2 "due to logistic limitations" - this completely undermines comparison of stress physiology between locations

Lines 142-143: "A stick was attached to each plant" - vague; no specification of stake height or tying method

Statistical analysis (Section 2.7): Why was Model 3 (AIC=373.04) even considered when Model 2 (AIC=291.88) was clearly superior?

Results

Table 3 - CRITICAL ERRORS: SPAD value for I0 shows 85.8 ± 17.66 - but this same value appears in Table 4 for Location 2 I0? Possible data copying errors

Table 3: LAI row for ID states "129.23 ± 14.3" but text says "highest LAI was measured under ID and lowest LAI was observed at ID" - contradictory/typo

Table 3: IC×MS shows SPAD 46.17 ± 4.19 (extremely low) while other IC combinations are higher - no explanation for this specific combination failing

Figure 3: Multiple panels (H2O2, MDA, CAT, APX, Proline) - figure quality is poor; letters for significance are barely visible

Section 3.3.1: Line 399 shows "I0 (22.27±3.63%)" at location 1 - but rainfed treatment should have lowest moisture, yet IC (27.62%) and ID (27.36%) are similar. ID should have higher moisture than IC if designed properly

Section 3.4: Figure 7 shows ID×MP at location 1 yielding ~15 t ha⁻¹, but Table 5 (IWUE) shows ID×Mp = 16.56 kg ha⁻¹ mm⁻¹ - these numbers don't align with yield calculations

Line 463: "IC×MP produced highest yield (23.18±6.62) at location 1, statistically similar with ID×MS (12.93±4.53)" - a difference of 10 t ha⁻¹ cannot be "statistically similar" unless test is underpowered

Discussion

Lines 532-536: Claim that "SPAD value was higher in non-irrigated plot compared to drip irrigation... due to smaller, thicker leaves" - but no leaf thickness or specific leaf area data to support this mechanism

Lines 550-554: Citing studies showing 14-50% IWUE improvement with drip irrigation - but location 1 showed drip underperforming conventional irrigation. This contradiction needs direct address

Lines 576-580: "H2O2 values at location 1 suggests that drip irrigation was not comparable" - but H2O2 was NOT measured at location 1 (only location 2). This is a fatal overreach

Lines 595-597: "Drip irrigation increased ~117% and ~32% fruit yield compared to no irrigation and conventional furrow at location 2" - but Table 4 shows ID (21.88) vs IC (14.68) = 49% increase, not 32%

Lines 610-614: The LMM interpretation overstates novelty - mixed models for agricultural field trials are standard

Tables and Figures

Table 1: Unit for EC is "S cm⁻¹" - should be "µS cm⁻¹" (microsiemens)

Table 1: Potassium units "meq 100-g⁻¹ soil" - non-standard; should use cmol kg⁻¹

Table 3 & 4: Significance markers (*, **, NS) - inconsistent use; no explicit legend explaining p-value thresholds

Figure 1: Poor quality map; location boundaries unclear

Figure 2: Weather data - temperature ribbon shows max and min, but rainfall data is missing from the figure despite being discussed

Figures 4,5,6,7: All use identical axis labels and formatting; letters for significance are too small and sometimes overlapping

Supplementary Figure 1: DHARMa diagnostic plots - the QQ plot shows some deviation at tails

References

Reference 34 (BARC, 2018) - fertilizer recommendation guide - no specific page or table cited

References 35-39 - methods papers from 1973-2021 - extraction protocols are not fully described in Methods, only cited

Reference 40 - "R Core Team, 2024" - incomplete; R version 4.4.1 has specific release date missing

Missing citations for agronomic practices in brinjal (plant spacing, fertilizer rates specific to eggplant)

Language and Presentation

Subject-verb agreement error in title: "Responses...depends"

Line 48: "Brinal" typo

Line 110: "location 2" (should be location 1) - typo

Inconsistent terminology: "conventional alternate furrow irrigation" vs "conventional light furrow irrigation"

Redundancy: "approximately ~10.6 t ha⁻¹" (Line 22)

Awkward phrasing: Line 456-457 "At location 2 the trend was found to be opposite, irrigation significantly boosted"

Missing units: Line 151 "55 mL of water" - per what time?

Overall Verdict Comments

Data inconsistencies between text, tables, and figures

Incomplete methodological reporting (irrigation amounts, stress marker protocols)

Critical design limitation (stress markers at only one location)

Overstated conclusions not supported by the data

Reproducibility is low due to insufficient detail on irrigation systems and protocols

**********

what does this mean?). If published, this will include your full peer review and any attached files.

If you choose “no”, your identity will remain anonymous but your review may still be made public.

**Do you want your identity to be public for this peer review?**  For information about this choice, including consent withdrawal, please see our Privacy Policy

**********

[NOTE: If reviewer comments were submitted as an attachment file, they will be attached to this email and accessible via the submission site. Please log into your account, locate the manuscript record, and check for the action link "View Attachments". If this link does not appear, there are no attachment files.]

To ensure your figures meet our technical requirements, please review our figure guidelines: https://journals.plos.org/plosone/s/figures

You may also use PLOS’s free figure tool, NAAS, to help you prepare publication quality figures: https://journals.plos.org/plosone/s/figures#loc-tools-for-figure-preparation.

NAAS will assess whether your figures meet our technical requirements by comparing each figure against our figure specifications.

Attachment Submitted filename: Peer review report-PONE-D-26-17291-7-5-2026.docx

# Author comment: Author response to Decision Letter 1

**DOI:** 10.1371/journal.pone.0360413.r002

7 Jul 2026

Dear Editorial Team,

We are pleased to resubmit the revised version of the above-mentioned manuscript for further consideration in PLOS ONE. We sincerely thank the editor, reviewer, and journal staff for their careful evaluation and constructive comments, which have helped us improve the scientific accuracy, clarity, transparency, and presentation of the manuscript.

We have addressed all reviewer, editor, and technical requirements. The revised submission includes:

1. A clean version of the revised manuscript with all changes accepted;

2. A marked-up version showing all tracked changes;

3. A detailed point-by-point response to the reviewer’s, editor’s, and journal requirements;

4. The minimal dataset underlying the reported results as Supporting Information; and

5. The analysis code, where applicable, as Supporting Information.

The required declarations are provided below.

Funding statement

This research was financially supported by the Australian Centre for International Agricultural Research (ACIAR) through the RUPANTAR project (WAC/2020/148, “Transforming smallholder food systems in the Eastern Gangetic Plains”). No additional internal funding was received. There was no additional external funding received for this study.

Role of the funder

The funders had no role in study design, data collection and analysis, decision to publish, or preparation of the manuscript.

Competing interests

The authors have declared that no competing interests exist.

Data availability

All data underlying the findings reported in this manuscript are provided in the manuscript and its Supporting Information files. The uploaded minimal dataset contains the replicate-level values used to calculate the reported means, standard deviations, figures, irrigation water-use efficiency estimates, and statistical analyses, together with the necessary variable descriptions and metadata.

Code availability

No author-generated software or custom code underpins the findings of this study. The analyses were conducted using ‘R’ (version 4.4.1) with standard statistical procedures described in the Materials and Methods section.

Figure and copyright statement

Figure 1 was redrawn by the authors using OpenStreetMap data. The figure does not contain Google Maps, Google Earth, or other proprietary map or satellite imagery. Appropriate OpenStreetMap attribution and licensing information have been included in the figure and its caption.

No copyrighted third-party figures or images are included in the manuscript.

Funding-related text has been removed from the Acknowledgments section, and the amended funding and funder-role statements are provided above for updating the online submission record.

We believe that all journal requirements have now been addressed. We appreciate the opportunity to revise the manuscript and thank you for considering it for publication in PLOS ONE.

Attachment Submitted filename: Response to Reviewers.docx

# Peer review: Decision Letter 1

**DOI:** 10.1371/journal.pone.0360413.r003

**Authors:** Umesh Sharma

2 Sep 2026

Thank you for submitting your manuscript to PLOS One. After careful consideration, we feel that it has merit but does not fully meet PLOS One’s publication criteria as it currently stands. Therefore, we invite you to submit a revised version of the manuscript that addresses the points raised during the review process.

Please submit your revised manuscript by Oct 17 2026 11:59PM. If you will need more time than this to complete your revisions, please reply to this message or contact the journal office at plosone@plos.org. When you're ready to submit your revision, log on to https://www.editorialmanager.com/pone/ and select the 'Submissions Needing Revision' folder to locate your manuscript file.

- A letter that responds to each point raised by the academic editor and reviewer(s). You should upload this letter as a separate file labeled 'Response to Reviewers'.
- A marked-up copy of your manuscript that highlights changes made to the original version. You should upload this as a separate file labeled 'Revised Manuscript with Track Changes'.
- An unmarked version of your revised paper without tracked changes. You should upload this as a separate file labeled 'Manuscript'.

If you would like to make changes to your financial disclosure, please include your updated statement in your cover letter. Guidelines for resubmitting your figure files are available below the reviewer comments at the end of this letter.

If applicable, we recommend that you deposit your laboratory protocols in protocols.io to enhance the reproducibility of your results. Protocols.io assigns your protocol its own identifier (DOI) so that it can be cited independently in the future. For instructions see: https://journals.plos.org/plosone/s/submission-guidelines#loc-laboratory-protocols. Additionally, PLOS One offers an option for publishing peer-reviewed Lab Protocol articles, which describe protocols hosted on protocols.io. Read more information on sharing protocols at https://plos.org/protocols?utm_medium=editorial-email&utm_source=authorletters&utm_campaign=protocols.

As the corresponding author, your ORCID iD is verified in the submission system and will appear in the published article. PLOS supports the use of ORCID, and we encourage all coauthors to register for an ORCID iD and use it as well. Please encourage your coauthors to verify their ORCID iD within the submission system before final acceptance, as unverified ORCID iDs will not appear in the published article. *Only*  the individual author can complete the verification step; PLOS staff *cannot*  verify ORCID iDs on behalf of authors.

We look forward to receiving your revised manuscript.

Kind regards,

Umesh Sharma

Academic Editor

PLOS One

Journal Requirements:

If the reviewer comments include a recommendation to cite specific previously published works, please review and evaluate these publications to determine whether they are relevant and should be cited. There is no requirement to cite these works unless the editor has indicated otherwise.

Please review your reference list to ensure that it is complete and correct. If you have cited papers that have been retracted, please include the rationale for doing so in the manuscript text, or remove these references and replace them with relevant current references. Any changes to the reference list should be mentioned in the rebuttal letter that accompanies your revised manuscript. If you need to cite a retracted article, indicate the article’s retracted status in the References list and also include a citation and full reference for the retraction notice.

Additional Editor Comments:

Please incorporate all the comments recommended by reviewers and check complete MS for minor errors.

Reviewers' comments:

Reviewer's Responses to Questions

**Comments to the Author**

Reviewer #1: All comments have been addressed

Reviewer #2: (No Response)

**********

2. Is the manuscript technically sound, and do the data support the conclusions?

Reviewer #1: Yes

Reviewer #2: Yes

**********

3. Has the statistical analysis been performed appropriately and rigorously?

Reviewer #1: Yes

Reviewer #2: Yes

**********

4. Have the authors made all data underlying the findings in their manuscript fully available?

The PLOS Data policy

Reviewer #1: Yes

Reviewer #2: Yes

**********

5. Is the manuscript presented in an intelligible fashion and written in standard English?

Reviewer #1: Yes

Reviewer #2: Yes

**********

Reviewer #1: All corrections accepted

Review Verdict

Accept with Minor Revisions

Key Strengths

Robust experimental design and statistical analysis (LMM with proper model selection)

Important practical implications for smallholder irrigation in South Asia

Transparent acknowledgment of limitations

Remaining Minor Issues (4 items)

Abstract typo: "IC and IC" → "IC and I₀" (Line 17, Page 10)

LAI contradiction: Lowest LAI at Location 1 should be under I₀, not I_D (Line 313-317, Page 26)

Grammar: "At both the locations" → "At both locations" (Line 578, Page 95)

Grammar: "H₂O₂ values...suggests" → "suggest" (Line 638, Page 98)

Recommendation

Publish after correcting the 4 minor language/typo issues above.

Reviewer #2: This manuscript addresses a timely topic—screening resilient plants and waste-derived substrates for vertical greenery systems under combined drought and waterlogging stresses—and provides useful experimental data, particularly the identification of Buxus sinica as the top performer and the optimized 3:1:1 garden-waste:straw:biogas residue substrate. However, the study has several significant shortcomings that require major revision. The most critical issue is that the methodology describes only separate drought and waterlogging experiments, yet the abstract and introduction repeatedly emphasize "alternating stresses" as the core novelty—this disconnect must be clarified or the framing revised accordingly. Additionally, the claimed novelty of the "customized" fuzzy membership model is overstated without clear specification of what is improved; statistical reporting is insufficient (ANOVA results and significance indicators missing from figures); the drought treatment lacks explicit soil moisture thresholds; and the language contains numerous errors (e.g., inconsistent species names, "Draught" in Table 1). While the experimental design is generally sound and the substrate optimization has practical value, the manuscript needs thorough revision to address these issues, after which it could become a worthwhile contribution to the VGS literature

**********

what does this mean?). If published, this will include your full peer review and any attached files.

If you choose “no”, your identity will remain anonymous but your review may still be made public.

**Do you want your identity to be public for this peer review?**  For information about this choice, including consent withdrawal, please see our Privacy Policy

Reviewer #1: **Yes:**  Prof.Dr.Hani Mansour

Reviewer #2: No

**********

[NOTE: If reviewer comments were submitted as an attachment file, they will be attached to this email and accessible via the submission site. Please log into your account, locate the manuscript record, and check for the action link "View Attachments". If this link does not appear, there are no attachment files.]

To ensure your figures meet our technical requirements, please review our figure guidelines: https://journals.plos.org/plosone/s/figures

You may also use PLOS’s free figure tool, NAAS, to help you prepare publication quality figures: https://journals.plos.org/plosone/s/figures#loc-tools-for-figure-preparation.

NAAS will assess whether your figures meet our technical requirements by comparing each figure against our figure specifications.

# Author comment: Author response to Decision Letter 2

**DOI:** 10.1371/journal.pone.0360413.r004

2 Sep 2026

Dear Academic Editor and Reviewers,

We sincerely thank the Academic Editor and the reviewers for their careful evaluation of our revised manuscript. We appreciate the positive assessment of the revisions made during the previous round and the opportunity to address the remaining points.

All applicable comments have been carefully addressed in the revised manuscript. Our point-by-point responses are provided below.

Response to the Academic Editor

Comment:

Please incorporate all the comments recommended by reviewers and check complete MS for minor errors.

Response:

Thank you for the guidance. The complete manuscript has been carefully checked again for typographical, grammatical, and presentation-related errors. All four remaining minor corrections identified by Reviewer 1 have been incorporated into the revised manuscript. We have also carefully examined the comments attributed to Reviewer 2. As explained below, those comments appear to concern a different manuscript and therefore could not appropriately be incorporated into the present study.

Reviewer 1

We sincerely thank Reviewer 1 for reassessing our revised manuscript and for confirming that the previous comments have been adequately addressed. We also appreciate the identification of the four remaining minor issues. Reviewer 1 assessed the work as technically sound and statistically appropriate and recommended publication following these corrections.

Comment 1: Abstract typo: “IC and IC” → “IC and I₀” (Line 17, Page 10).

Response:

Thank you for identifying this typographical error. In fact, the drip irrigation outperformed the control (I0) by 117% and the conventional furrow irrigation (IC) to 32%. Therefore, in accoradnace with the result, the text has been corrected from “IC and IC” to “I0 and IC” in the Abstract.

Comment 2: LAI contradiction: Lowest LAI at Location 1 should be under I₀, not I_D (Lines 313–317, Page 26).

Response:

Thank you for identifying this inconsistency. The statement has been corrected to indicate that the lowest LAI at Location 1 was recorded under I₀, in agreement with the corresponding results.

Comment 3: Grammar: “At both the locations” → “At both locations” (Line 578, Page 95).

Response:

Thank you. The phrase has been corrected from “At both the locations” to “At both locations.”

Comment 4: Grammar: “H₂O₂ values...suggests” → “suggest” (Line 638, Page 98).

Response:

Thank you for identifying the grammatical error. The verb has been corrected from “suggests” to “suggest” to agree with the plural subject “H₂O₂ values.”

Reviewer 2

We thank Reviewer 2 for evaluating the manuscript. We note that Reviewer 2 answered “Yes” to the questions concerning technical soundness, appropriateness and rigor of the statistical analysis, availability of underlying data, and presentation in standard English.

Comment: The reviewer comments on screening resilient plants and waste-derived substrates for vertical greenery systems, Buxus sinica, a 3:1:1 garden-waste:straw:biogas-residue substrate, drought and waterlogging treatments, alternating stresses, and a fuzzy membership model.

Response

Thank you for the assessment. We have carefully examined this comment. However, we respectfully believe that the comment may have been inadvertently associated with the present manuscript, as the topics and experimental details described in the comment do not correspond to our study.

The present manuscript, entitled “Plastic Mulching Consistently Improves Brinjal Performance, but Drip Irrigation Benefits Are Soil Dependent: A Two-Location Field Study,” investigates the effects of plastic mulching and drip irrigation on brinjal under field conditions at two locations. It does not involve vertical greenery systems, Buxus sinica, waste-derived growth substrates, drought and waterlogging screening, alternating stress treatments, or fuzzy membership modelling. In contrast, all of these elements are specifically referred to in the comment attributed to Reviewer 2.

Consequently, we are unable to make scientifically appropriate revisions to the present manuscript on the basis of these comments because the issues raised do not relate to its objectives, experimental design, treatments, analyses, or findings.

We respectfully request the Academic Editor to consider this apparent discrepancy. If Reviewer 2 has any additional comments or recommendations specifically related to the present manuscript, we would be very pleased to receive them and will address them carefully and comprehensively.

We thank the reviewers and the Academic Editor for their consideration.

Attachment Submitted filename: Response to Reviewers_R2.docx

# Editor report: Decision Letter 2

**DOI:** 10.1371/journal.pone.0360413.r005

**Authors:** Umesh Sharma

28 Sep 2026

Plastic mulching consistently improves brinjal performance, but drip irrigation benefits are soil dependent: A two-Location field study

PONE-D-26-17291R2

Dear Dr. Rashid,

We’re pleased to inform you that your manuscript has been judged scientifically suitable for publication and will be formally accepted for publication once it meets all outstanding technical requirements.

Within one week, you’ll receive an e-mail detailing the required amendments. When these have been addressed, you’ll receive a formal acceptance letter and your manuscript will be scheduled for publication.

An invoice will be generated when your article is formally accepted. Please note, if your institution has a publishing partnership with PLOS and your article meets the relevant criteria, all or part of your publication costs will be covered. Please make sure your user information is up-to-date by logging into Editorial Manager at Editorial Manager® and clicking the ‘Update My Information' link at the top of the page. For questions related to billing, please contact billing support.

If your institution or institutions have a press office, please notify them about your upcoming paper to help maximize its impact. If they’ll be preparing press materials, please inform our press team as soon as possible -- no later than 48 hours after receiving the formal acceptance. Your manuscript will remain under strict press embargo until 2 pm Eastern Time on the date of publication. For more information, please contact onepress@plos.org.

Kind regards,

Dr. Umesh Sharma

Academic Editor

PLOS One

Additional Editor Comments (optional):

Dear Authors, please carefully check the entire proof of the manuscript, kindly pay particular attention to minor typographical and spelling errors, grammar and sentence structure, in-text citations and their consistency with the reference list, author names, publication years, and citation formatting, figure and table captions, numbering, and cross-references, units, symbols, numerical values, and statistical notation, scientific names and their formatting, headings and subheadings and any formatting inconsistencies introduced during typesetting.

Reviewers' comments:

# Editor report: Acceptance letter

**DOI:** 10.1371/journal.pone.0360413.r006

**Authors:** Umesh Sharma

PONE-D-26-17291R2

PLOS One

Dear Dr. Rashid,

I'm pleased to inform you that your manuscript has been deemed suitable for publication in PLOS One. Congratulations! Your manuscript is now being handed over to our production team.

At this stage, our production department will prepare your paper for publication. This includes ensuring the following:

* All references, tables, and figures are properly cited

* All relevant supporting information is included in the manuscript submission,

* There are no issues that prevent the paper from being properly typeset

You will receive further instructions from the production team, including instructions on how to review your proof when it is ready. Please keep in mind that we are working through a large volume of accepted articles, so please give us a few days to review your paper and let you know the next and final steps.

Lastly, if your institution or institutions have a press office, please let them know about your upcoming paper now to help maximize its impact. If they'll be preparing press materials, please inform our press team within the next 48 hours. Your manuscript will remain under strict press embargo until 2 pm Eastern Time on the date of publication. For more information, please contact onepress@plos.org.

You will receive an invoice from PLOS for your publication fee after your manuscript has reached the completed accept phase. If you receive an email requesting payment before acceptance or for any other service, this may be a phishing scheme. Learn how to identify phishing emails and protect your accounts at https://explore.plos.org/phishing.

If we can help with anything else, please email us at customercare@plos.org.

Thank you for submitting your work to PLOS One and supporting open access.

Kind regards,

PLOS One Editorial Office Staff

on behalf of

Dr. Umesh Sharma

Academic Editor

PLOS One
